# Stem-loop structures control mRNA processing of the cellulosomal *cip-cel* operon in *Ruminiclostridium cellulolyticum*

**DOI:** 10.1186/s13068-023-02357-5

**Published:** 2023-06-29

**Authors:** Na Wang, Ping Li, Ying Cheng, Houhui Song, Chenggang Xu

**Affiliations:** 1 Key Laboratory of Applied Technology on Green-Eco-Healthy Animal Husbandry of Zhejiang Province, China-Australia Joint Laboratory for Animal Health Big Data Analytics, Zhejiang Provincial Engineering Research Center for Animal Health Diagnostics & Advanced Technology, Zhejiang International Science and Technology Cooperation Base for Veterinary Medicine and Health Management, College of Animal Science and Technology & College of Veterinary Medicine, Zhejiang A&F University, Hangzhou, 311300 Zhejiang China; 2 Key Laboratory of Chemical Biology and Molecular Engineering of Ministry of Education, Institute of Biotechnology, Shanxi University, Taiyuan, 030006 Shanxi China

**Keywords:** *Ruminiclostridium cellulolyticum*, Cellulosome, The *cip-cel* operon, RNA processing, Stem-loop structure

## Abstract

**Background:**

Anaerobic, mesophilic, and cellulolytic *Ruminiclostridium cellulolyticum* produces an efficient cellulolytic extracellular complex named cellulosome, which consist of a non-catalytic multi-functional integrating subunit, organizing the various catalytic subunits into the complex. Main components of cellulosome were encoded by the *cip-cel* operon in *R. cellulolyticum*, and their stoichiometry is controlled by the mechanism of selective RNA processing and stabilization, which allows to confer each processed RNA portion from the *cip-cel* mRNA on different fates due to their stability and resolve the potential contradiction between the equimolar stoichiometry of transcripts with a within a transcription unit and the non-equimolar stoichiometry of subunits.

**Results:**

In this work, RNA processing events were found to occur at six intergenic regions (IRs) harboring stem-loop structures in *cip-cel* operon. These stem-loops not only stabilize processed transcripts at their both ends, but also act as cleavage signals specifically recognized by endoribonucleases. We further demonstrated that cleavage sites were often located downstream or 3′ end of their associated stem-loops that could be classified into two types, with distinct GC-rich stems being required for RNA cleavage. However, the cleavage site in IR4 was found to be located upstream of the stem-loop, as determined by the bottom AT-pair region of this stem-loop, together with its upstream structure. Thus, our findings reveal the structural requirements for processing of *cip-cel* transcripts, which can be potentially used to control the stoichiometry of gene expression in an operon.

**Conclusions:**

Our findings reveal that stem-loop structures acting as RNA cleavage signals not only can be recognized by endoribonucleases and determine the location of cleavage sites but also determine the stoichiometry of their flanking processed transcripts by controlling stability in *cip-cel* operon. These features represent a complexed regulation of cellulosome in the post-transcriptional level, which can be exploited for designing synthetic elements to control gene expression.

**Supplementary Information:**

The online version contains supplementary material available at 10.1186/s13068-023-02357-5.

## Introduction

*Ruminiclostridium cellulolyticum* (previously *Clostridium cellulolyticum*), a cellulosome-producing and anaerobic cellulolytic Gram-positive bacterium, is one research model organism and a promising host for biofuel production from lignocellulose [1–4]. The cellulosome is an extracellular enzymatic complex, and represents a significant model for the efficient biological degradation of cellulosic biomass [5–7]. The *cip-cel* operon in *R. cellulolyticum* harbors 12 genes that encode the major cellulosomal subunits that are essential for cellulose degradation. Transcriptional analysis of the *cip-cel* operon showed several transcripts displaying different stabilities were produced suggesting the processing of *cip-cel* mRNA [8]. Our data of differential RNA-Seq confirmed that *cip-cel* mRNA was processed initially into several segments, and then, variation in the stability between these segments contributed to their differential expression [9].

In bacteria, numerous mRNAs are processed to mature forms like rRNAs and tRNAs, and this is a key event regulating these genes at the post-transcriptional level, often in response to environmental constraints. During the process of RNA maturation, RNases and small RNAs (sRNAs) that would otherwise promote RNA degradation convert labile RNAs into stable and biologically functional molecules [10]. Compared to transcriptional regulation, which involves RNA synthesis, post-transcriptional processing provides a manner of regulation that saves time and energy relative to de novo RNA synthesis. A number of Gram-negative bacterial operons have been reported to undergo RNA processing, such as *rpsU-dnaG-rpoD* [11, 12], *glmU-glmS* [13], and *LEE4* [14] in *Escherichia coli*, and *cagA* [15] in *Helicobacter pylori*. In the gram-positive *Bacillus subtilis*, the bicistronic mRNA of the glycolysis operon *cggR-gapA* was shown to be cleaved near the end of *cggR* resulting in an unstable *cggR* portion and a stable *gapA* portion [16, 17]. Similar results have also been found in other gram-positive bacteria, such as *cdd-bmpA* [18], and *speB* [19] in *Streptococcus pyogenes* and *saePQRS* [20] in *Staphylococcus aureus*. As a general rule, RNA processing is initiated via primary cleavage by an endoribonuclease, leading to the generation of decay intermediates, and variation in stability in the segments generated by exoribonuclease contributes to differential gene expression.

In *E. coli* and related Gram-negative species, initiating RNA processing is mainly accomplished by the endoribonuclease RNase E, which is essential for cell viability [21, 22] and which cleaves predominately in AU-rich single-stranded regions [14, 23–25]. However, in the Gram-positive phylum of Firmicutes (e.g., *B. subtilis* and *S. aureus*) where RNase E is absent, RNase Y has been proposed to be the enzyme responsible for the cleavage of bulk mRNAs and to be the functional equivalent of RNase E [26]. It has been shown that RNase Y in *Streptococcus pyogenes* and *Staphylococcus aureus* prefers to cleave downstream of a G based on RNA-seq data. And RNase Y was found to cleave in single-stranded regions close to secondary structures, such as the *saePQRS* operon in *S. aureus* [18, 20, 27]. Recently, *E. coli* RNase E was shown to almost completely restore wild-type growth in an *rny* mutant of *B. subtilis* [26], suggesting that these two enzymes share similar in-vivo cleavage specificities. In addition, the exclusive endoribonucleases activity of RNase J in *B. subtilis* has been shown to initiate decay of the small *trp* leader RNA by cleaving the upstream of the 3′ transcription terminator [28, 29] and also to cut away the leader region from the *thrS* mRNA, causing an increase in downstream *thrS* mRNA stability [30–34]. Although endoribonucleases play a key role in initiating the degradation associated with mRNA processing, our understanding of their structures, enzymatic activities and sequence requirements is still in its infancy.

The aim of this work was to elucidate the sequence/structural requirements of processing for *cip-cel* transcripts. Here, we show that RNA cleavage events happen at six intergenic regions harboring stem-loop structures in the *cip-cel* operon and the secondary structures at both ends of processed transcripts contribute to their stability. Cloning analysis revealed that all cleavage of *cip-cel* mRNAs was determined by their nearby stem-loops, which included two distinct types of stem sequences. Thus, our study shows that the processing of *cip-cel* transcripts is controlled by stem-loop structures that can be recognized by endoribonucleases and determine the location of the cleavage sites.

## Results

### Transcripts of the *cip-cel* operon cleaved in six intergenic regions

**Fig. 1:**
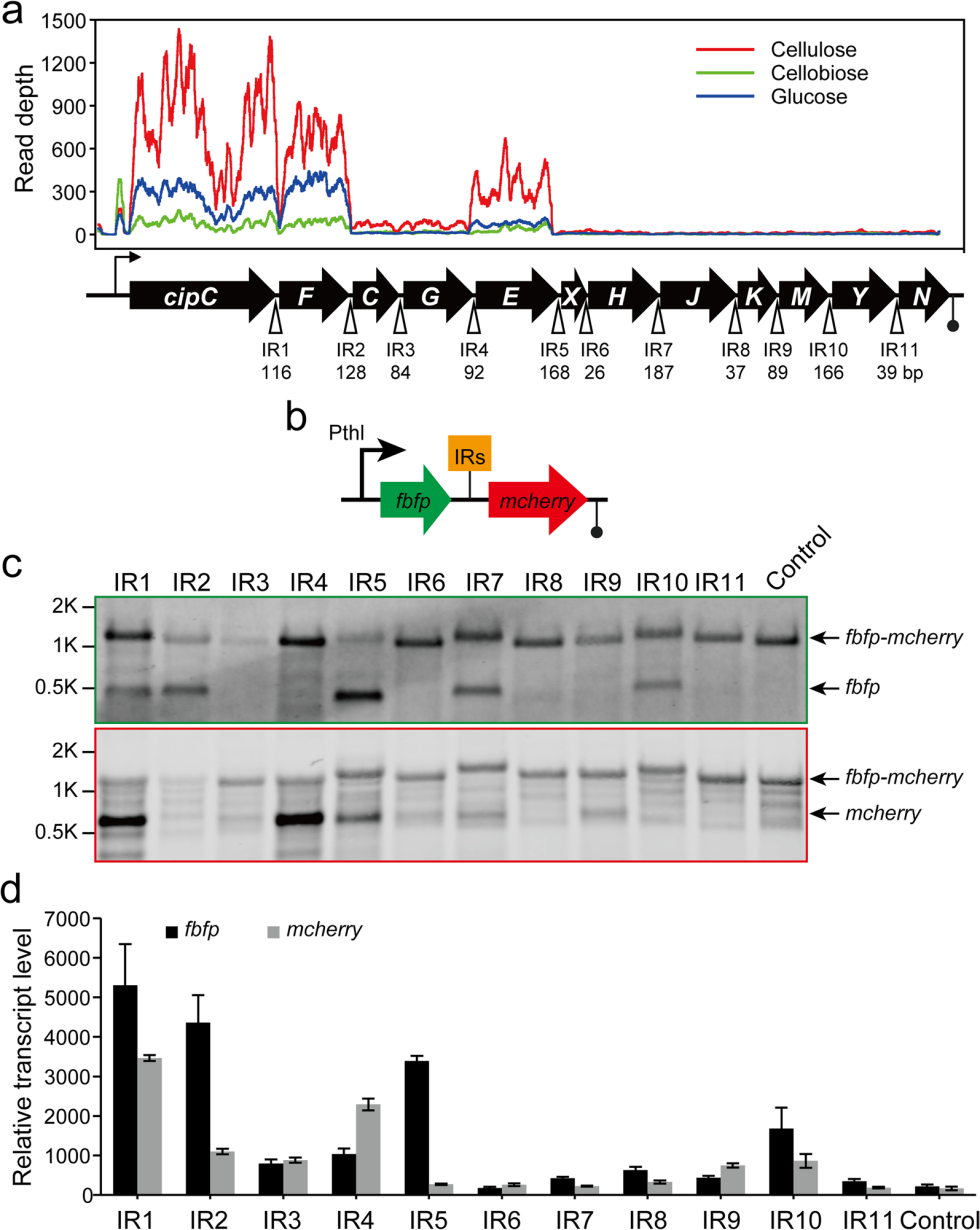
Differential transcription of genes in the *cip-cel* operon initiated by RNA cleavage. **a** Transcriptional profiles of the *cip-cel* operon grown on glucose, cellobiose and cellulose. Eleven intergenic regions (IRs) are indicated by their length. **b** Schematic representation of dual-fluorescence reporter system for analysis of RNA cleavage, where IRs were inserted between *fbfp* and *mcherry*. **c** Northern blotting analysis of transcripts using the dual-fluorescence reporter system carrying each IR with *fbfp*- and *mcherry*-targeting probes. Black arrows highlight the positions of bands that corresponded to transcripts, as indicated on the right side of the panel. **d** Relative transcription level of *fbfp* and *mcherry* in artificial operons harboring various IRs, as measured by qRT-PCR. Data were normalized via the transcript level of the gene of *Ccel_RS01560*, encoding the RNA polymerase beta subunit. The artificial operon harboring no IRs was used as a control. Error bars indicate the standard deviation of the mean from experiments done in triplicate

The *cip-cel* operon spanning 26 kb in the genome of *R. cellulolyticum* harbors 12 genes that encode the major cellulosomal subunits that are essential for cellulose degradation. Gene *cipC*, encoding a cellulosomal scaffolding, is the first gene of this operon and is followed by 11 other genes (*cel48F*, *cel8C*, *cel9G*, *cel9E*, *orfX*, *cel9H*, *cel9J*, *man5K*, *cel9M*, *rgl11Y*, and *cel5N*), most of which encode cellulases (Fig. 1a). Our previous transcriptome data revealed that the primary *cip-cel* mRNA was selectively processed at several intergenic regions (IRs) between *cipC* and *cel48F*, *cel48F* and *cel8C*, *cel9G* and *cel9E*, and *cel9E* and *orfX*, resulting in secondary transcripts having different abundances due to their difference in stability (Fig. 1a) [9]. To further determine the RNA processing events in the *cip-cel* operon, each of the 11 IRs of the *cip-cel* operon (consecutively numbered IR1-IR11, ranging from 26 to 187 bp; Additional file 1: Table S3) were, respectively, inserted between the reporter genes *fbfp* (encoding a green fluorescence protein) and *mcherry* (encoding a red fluorescence protein) (Fig. 1b). The resulting artificial operons were then separately introduced into *R. cellulolyticum* and the *fbfp-mcherry* reporter system without any inserted segments was used as a control.

To determine the in-vivo cleavage sites in the *cip-cel* mRNA, two probes were designed to detect transcripts that respectively harbored *fbfp* and *mcherry* by Northern blotting. Similar to the control that harboured no IRs, IR3, IR6, IR8, IR9 and IR11 exhibited only a single > 1.2-kb band (consistent with the expected size of the bicistronic transcript from *fbfp-mcherry*) detected by both probes, suggesting that they were not processed (Fig. 1c). In contrast, monocistronic transcripts of *fbfp* and/or *mcherry* were detected from cells with the artificial operons harboring IR1, IR2, IR4, IR5, IR7, and IR10, in addition to the full-size band of the bicistronic transcript, suggesting that there were RNA cleavage events occurring when these IRs were present. However, these RNA cleavage events appeared to have three consequences. First, both monocistronic transcripts of *fbfp* and *mcherry* were detected in IR1 and IR5. Second, only the upstream monocistronic transcript of *fbfp* was detected in IR2, IR7, and IR10. Finally, only the downstream monocistronic transcript of *mcherry,* was detected in IR4 (Fig. 1c).

Furthermore, the transcript abundance of *fbfp* and *mcherry* flanking IRs was measured by quantitative qRT-PCR (Fig. 1d). Compared with the control without any inserted segments, the relative transcript abundance of *fbfp* and *mcherry* from unprocessed IRs was only marginally different. However, the relative transcript abundance of *fbfp* and/or *mcherry* from processed IRs was significantly increased. For example, both *fbfp* and *mcherry* from IR1 were 24 and 21 times higher, respectively, compared with the controls. Moreover, the transcript abundances of *fbfp* from IR2, IR5 and IR10 and *mcherry* from IR4 were also significantly increased. The expression pattern of *fbfp* and *mcherry* in the report system harboring various IRs is almost consistent with the transcriptional profile of genes flanking IRs in the *cip-cel* operon except IR10 (Fig. 1a). Altogether, RNA cleavage occurred in six IRs of the *cip-cel* operon, resulting in significant differences in transcript abundance between upstream and downstream transcripts from cleavage sites.

### Stability of processed transcripts determined by the secondary structures at both ends

**Fig. 2:**
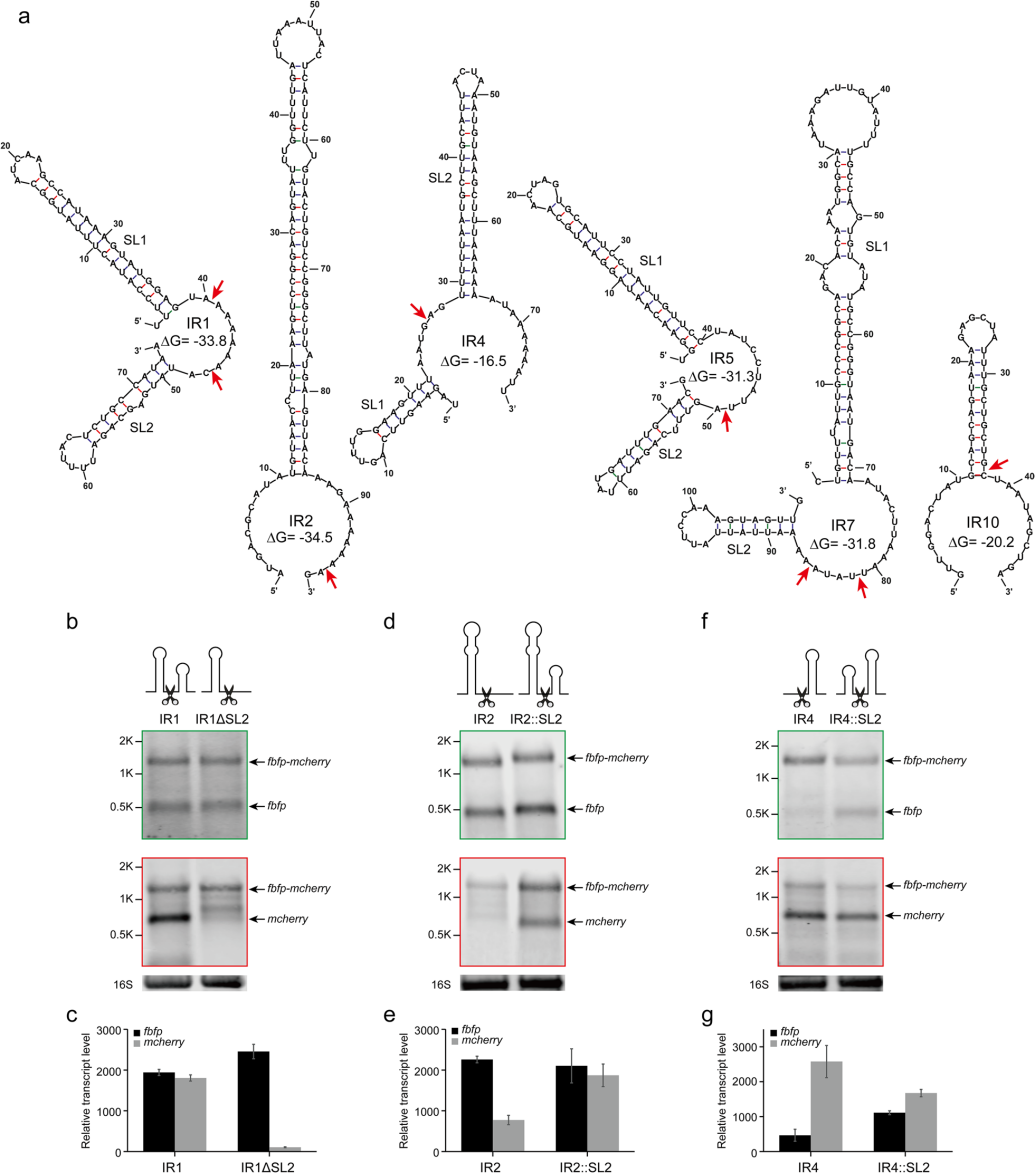
Stability of processed transcripts of the *cip-cel* operon determined by stem-loops at both ends. **a** Secondary structure prediction of IRs harboring RNA cleavage sites. The cleavage sites identified by primer extension are indicated by red arrows. **b**, **c** Effect of deletion of SL2 in IR1 using dual-fluorescence reporter system, as analyzed by Northern blotting (**b**) and qRT-PCR (**c**). **d**–**g** Effect of the addition of SL2 into IR2 and IR4, as analyzed by Northern blotting (**d**, **f**) and qRT-PCR (**e**, **g**). Black arrows highlight the positions of bands that correspond to transcripts, as indicated on the right side of the panel. 16S rRNA was used as a loading control. Error bars indicate the standard deviation of the mean from experiments done in triplicate

To gain further insight into RNA processing in the presence of these IRs, their secondary structures were analyzed and compared using Mfold [35, 36] (Additional file 1: Fig. S1). The prediction results of secondary structures indicated that the folding energy (ΔG) of IRs occurring cleavage events is much lower than that of IRs without cleavage, because each of them harbours a large stem-loop structure (ΔG < − 15 kcal mol^−1^, Fig. 2a). Thus, it suggested that processing of the *cip-cel* mRNA could be linked to the stem-loop structures located in its IRs. However, there were huge differences in the stem-loop structures of these different IRs. In this context, IR1, IR5, IR4 and IR7 harbored two consecutive stem-loop structures in which a small stem-loop was located upstream or downstream of a large stem-loop, while IR2 and IR10 had only one stem-loop. Stem-loops of IR1-SL1, IR5-SL1, and IR10-SL were complete in structure, while the stem-loops of IR2-SL and IR7-SL1 harbored some unpaired regions at the top and bottom, with a 6-GC pair core region in the middle of these stems (Fig. 2a).

Furthermore, RNA cleavage sites in the IRs were precisely identified by primer extension assay using a Cy5.5 5′-end labeled oligonucleotide specific to *mcherry*. These results indicated that cleavage sites were present near stem-loops and that the sequences flanking the cleavage sites were not conserved. Specifically, the cleavage sites in IR1, IR2, IR5, IR7, and IR10 were located the downstream or 3′ end of the large stem-loops, while that of IR4 was located upstream of its stem-loop (Additional file 1: Fig. S2). Remarkably, a small stem-loop structure is on the other side of the cleavage sites in IR1, IR4, IR5 and IR7 (Fig. 2a). This arrangement served to explain the differences in RNA cleavage patterns observed by Northern blotting (Fig. 1c) and indicated that they were due to differences in the secondary structure of processed transcript ends where stem-loops were involved in the protection of transcripts from exoribonuclease, resulting in their increased stability. For IR1 and IR5, the processed monocistronic transcripts of *fbfp* and *mcherry*, respectively, harbored a stem-loop in their 3′ and 5′ ends, resulting in both processed *fbfp* and *mcherry* being protected against exoribonuclease and becoming detectable by Northern blotting (Fig. 1c). In contrast, for IR2, IR7 and IR10, only processed monocistronic transcripts of *fbfp* harbored the large stem-loop at their 3′ end, indicating that only processed *fbfp* was protected, as could be detected by Northern blotting, while processed *mcherry* was degraded by exoribonuclease and was only marginally detectable, because the secondary structure at its 5′ end was not enough to protect it against exoribonuclease. On the contrary, processed *mcherry* was protected in IR4, while processed *fbfp* was degraded although it had a small stem-loop (Fig. 1c). Thus, these results suggest that there was 5′-to-3′ exoribonuclease activity in *R. cellulolyticum*, in addition to the 3′-to-5′ exoribonuclease activity similar to that of *E. coli*.

To test the effect of the secondary structure against exoribonuclease activity, we first added the second small stem-loop from IR1 (IR1-SL2) upstream of the reporter gene *fbfp* to analyze the stability of the transcript. This indicated that the addition of a stem-loop could significantly increase the half-life of *fbfp* transcripts from 5.3 to 45.3 min (Additional file 1: Fig. S3). In contrast, to further analyze the effect of stem-loops on processed transcripts, stem-loops were deleted or added near cleavage sites in IR1, IR2, and IR4. First, the secondary stem-loop of IR1 (named SL2) was deleted (named IR1ΔSL2), resulting in processed *mcherry* without any stem-loop at its 5′ end. The results from Northern blotting indicated that the monocistronic transcript of *fbfp* was still detectable in addition to the full-size band of the biocistronic transcript, while a few monocistronic transcripts of *mcherry* were detectable in IR1ΔSL2 (Fig. 2b). Moreover, our qRT-PCR results also confirmed that deletion of SL2 in IR1 significantly reduced the transcript level of *mcherry* (Fig. 2c). Thus, it appeared that deletion of SL2 in IR1 did not affect its processing but rather reduced the stability of the processed *mcherry* transcript, resulting in a much lower abundance of *mcherry*. In contrast, SL2 was respectively added downstream of the cleavage site in IR2 (named IR2::SL2) and upstream of IR4 (named IR4::SL2), resulting in processed *mcherry* from IR2 with SL2 at its 5′ end or processed *fbfp* from IR4 with SL2 at its 3′ end. We found processed monocistronic transcripts of *mcherry* from IR2 and *fbfp* from IR4 that could not be detected by Northern blotting, but were clearly detected after the introduction of SL2 (Fig. 2d, f), which was also confirmed by qRT-PCR (Fig. 2e, g), suggesting that the addition of SL2 to the ends of processed transcripts could significantly increase their stability. Thus, the processed transcripts of the *cip-cel* operon in *R. cellulolyticum* were degraded at both the 5′ and 3′ ends, and could be protected through the introduction of RNA secondary structures at their ends.

### Stem-loops are involved in the processing of the *cip-cel* mRNA

**Fig. 3:**
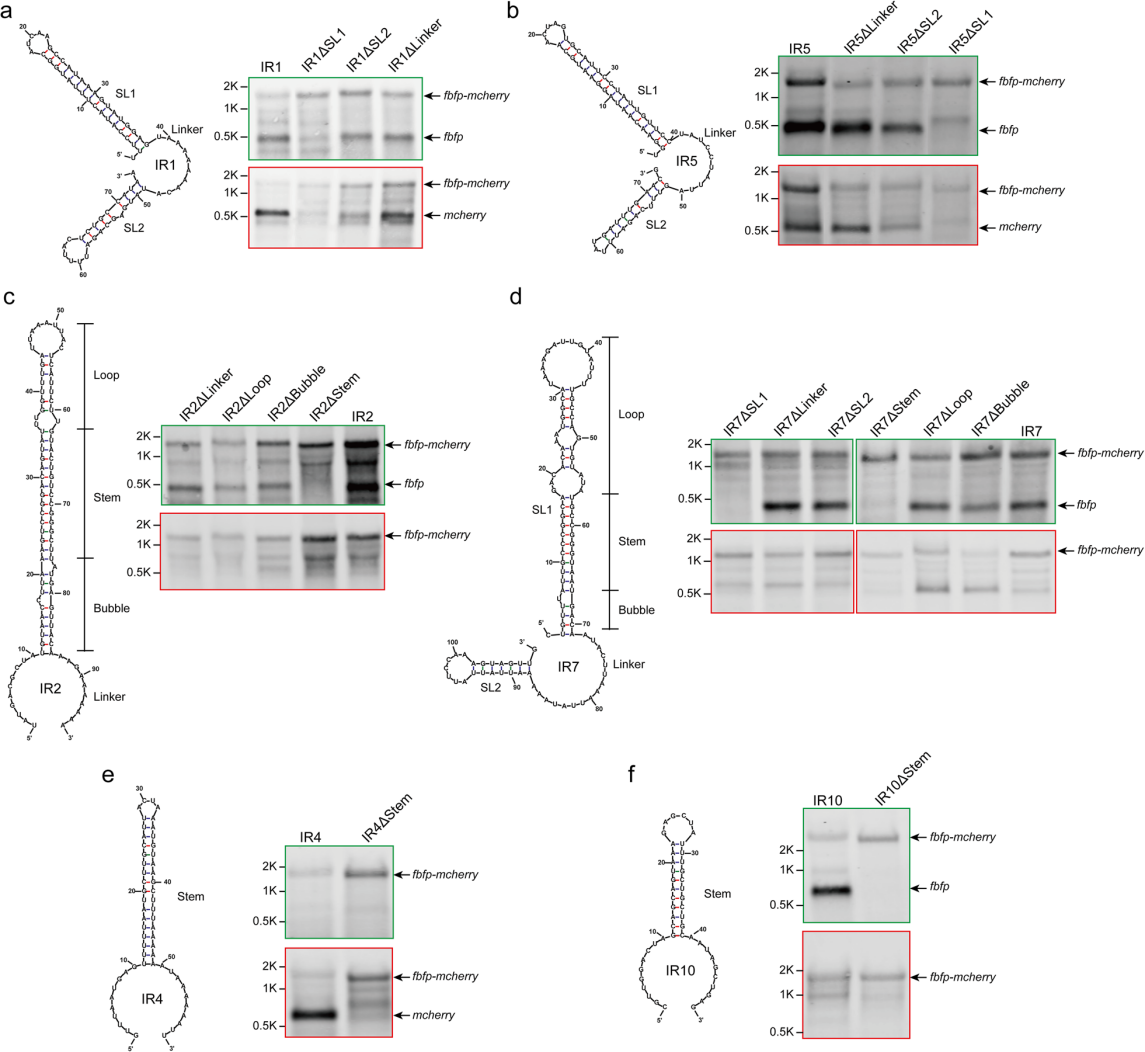
Functional analysis of secondary structures predicted in IRs harboring cleavage sites. The secondary structures of IR1 (**a**), IR5 (**b**), IR2 (**c**), IR7 (**d**), IR4 (**e**), and IR10 (**f**) were, respectively, mutated and inserted into the dual-fluorescence reporter system. Transcripts were analyzed by Northern blotting using *fbfp*- and *mcherry*-targeting probes. Black arrows highlight the positions of bands that correspond to transcripts as indicated on the right side of the panel. 16S rRNA was used as a loading control

To further define the sequence or structural requirements for *cip-cel* mRNA processing, we constructed three IR1 derivatives by, respectively, deleting their two stem-loops and the linker between them (named IR1∆SL1, IR1∆SL2, and IR1∆linker). These constructs were also inserted into our *fbfp* and *mcherry* reporter system, and processing was analyzed by Northern blotting (Fig. 3a). Interestingly, the deletion of the 37-bp linker encompassing the cleavage site (IR1∆linker) and its downstream small stem-loop (IR1∆SL2) did not prevent cleavage of IR1. In contrast, the cleavage no longer occurred when the stem-loop upstream of this cleavage site was deleted (IR1∆SL1). Similar results were found using IR5 or IR7 in which the stem-loop upstream of the cleavage site (SL1) determined the processing of IR5 or IR7 but not the linker harboring a cleavage site or its downstream SL2 (Fig. 3b, d). This indicated that the cleavage of IR1, IR5 and IR7 required a specific stem-loop structure that resulted in a cleavage site at a specific distance downstream of a transcript.

Furthermore, compared to IR1 and IR5, the stem-loop structures from IR2 and IR7 harbored many unpaired regions in their stems. These could be divided into three regions including a top loop, a middle-paired stem, and a bottom bubble. These three regions were, respectively, deleted to test their effect on cleavage. Results from Northern blotting showed that the cleavage between *fbfp* and *mcherry* was not blocked until the stem regions were deleted in both cases (Fig. 3c, d). Unsurprisingly, for IR4 and IR10, these stem-loop structures were also crucial to their cleavage, although their cleavage sites were respectively located upstream and downstream of their stem-loops (Fig. 3e, f). Altogether, the above findings suggested that *cip-cel* processing was determined by stem-loop structures harbored in their IRs. However, these stem-loops were not composed of certain conserved sequences and were vastly different from each other in structure.

### GC-rich regions of Type I stem-loops crucial for RNA cleavage

The stem-loops involved in RNA cleavage can be classified into three groups according to the characteristics of their structures (Fig. 2a). These are: Type I of IR1-SL1, IR5-SL1, and IR10-SL, which have a complete stem; Type II of IR2-SL and IR7-SL1, which have many unpaired regions; and Type III of IR4-SL2, of which the bottom is made of many AU links.

**Fig. 4:**
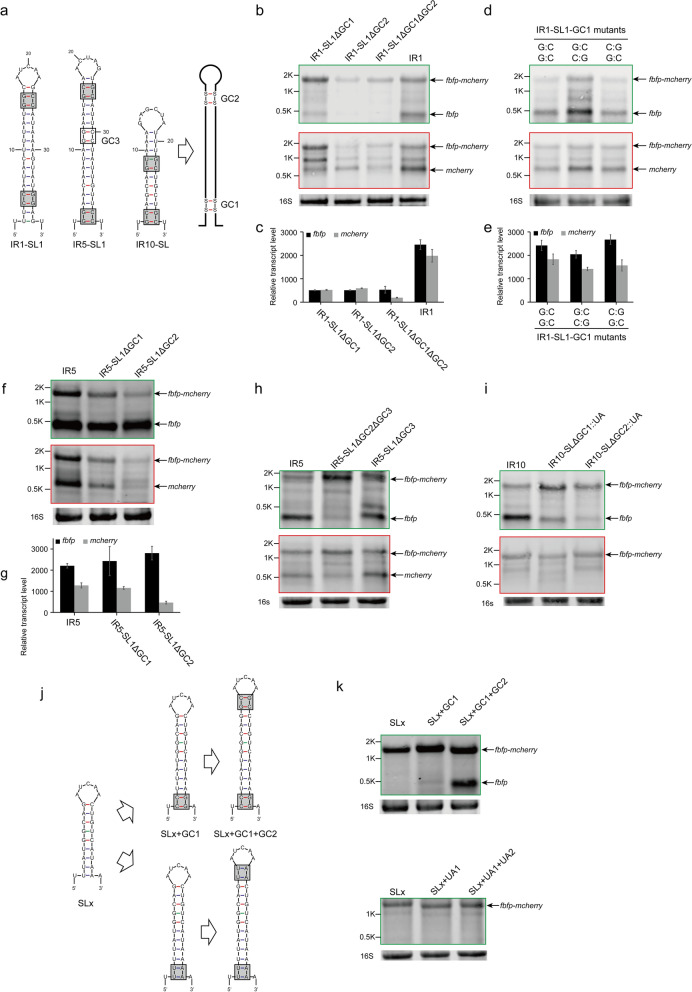
Structural requirements of Type I stem-loops for RNA cleavage. **a** Analysis of the structural conservation of the Type I stem-loops located in IR1, IR5, and IR10, in which there are two 2-GC-pair regions, respectively, located at the bottom (GC1) and top (GC2) of stems. **b**, **c** Effect of deletion of the 2-GC-pair region of IR1-SL1 in the dual-fluorescence reporter system, as analyzed by Northern blotting (**b**) and qRT-PCR (**c**). **d**, **e** Effect of the arrangement of GC1 in IR1-SL1, as analyzed by Northern blotting (**d**) and qRT-PCR (**e**). **f**, **g** Effect of deletion of the 2-GC-pair region of IR5-SL1, as analyzed by Northern blotting (**f**) and qRT-PCR (**g**). **h**, **i** Effect of deletion of the GC3 of IR5-SL1 (**h**) and replacement of two 2-GC-pair region of IR10-SL (**i**) was analyzed by Northern blotting. **j**, **k** Stem-loop of Type I was designed employing the stem-loop SLx (**j**) and its function was analyzed by Northern blotting (**k**). Black arrows highlight the positions of bands that correspond to transcripts as indicated on the right side of the panel of Northern blotting results. 16S rRNA was used as a loading control. Error bars indicate the standard deviation of the mean from experiments done in triplicate

We found that IR1-SL1, IR5-SL1, and IR10-SL of Type I harbored two 2-GC-pair regions that were located at the top and bottom, respectively, of their stems (named GC1 and GC2, respectively), which were potentially key sequences for RNA cleavage (Fig. 4a). To verify our hypothesis, two 2-GC-pair regions from IR1-SL1 were deleted,the results of Northern blotting indicated that the deletion of the GC1 (named IR1-SL1ΔGC1) or GC2 (named IR1-SL1ΔGC2) region led to a significant reduction in the cleavage efficiency and transcript level of *fbfp* and *mcherry*. When both were deleted (named IR1-SL1ΔGC1ΔGC2), the RNA cleavage was completely abolished (Fig. 4b, c). However, changing the order of the GC pairs in GC1 had no effect on the RNA cleavage and transcript level of *fbfp* and *mcherry* (Fig. 4d, e). Thus, it appeared that both 2-GC-pair regions of IR1-SL1 played a crucial role in RNA cleavage of IR1-SL1, while they did not appear to exhibit sequence specificity.

Furthermore, two 2-GC-pair regions of IR5-SL1 were also, respectively, deleted. The results from Northern blotting showed that the abundance of downstream monocistronic transcripts of *mcherry* was significantly decreased when both 2-GC-pair regions, especially GC2, were deleted, suggesting that both 2-GC-pair regions of IR5-SL1 were important for RNA cleavage, similar to IR1-SL1. However, the abundance of upstream monocistronic transcripts of *fbfp* was hardly changed when the 2-GC-pair regions were deleted (Fig. 4f). Meanwhile, the results of qRT-PCR indicated that the transcript level of *mcherry* was significantly decreased when the 2-GC-pair regions were deleted, while the levels of *fbfp* were unaffected (Fig. 4g). Thus, these results suggested that IR5-SL1 was not only the processing signal recognized by endoribonucleases, but also functioned as an internal rho-independent terminator, resulting in premature transcription termination after *fbfp*. Moreover, it appeared that GC2 was crucial for RNA cleavage, but not for transcription termination. However, there is another 2-GC-pair region in the middle of IR5-SL1 (named GC3) in addition to two 2-GC-pair regions at the top and bottom. It was showed that the deletion of the GC3 (named IR5-SL1∆GC3) did not prevent cleavage of IR5, but when it was deleted together with GC2 (named IR5-SL1∆GC2∆GC3), the abundance of monocistronic transcripts of *fbfp* was significantly decreased, suggesting that GC3 is important to transcription termination (Fig. 4h). It is consistent with the structure of IR5-SL1, which harbored a 3′ U-rich tract and is predicted to be a potential rho-independent terminator by TransTermHP [37]. These results also explained why the five genes located upstream of IR5 from the *cip-cel* operon are transcribed at higher levels than the downstream seven genes, as shown in the transcriptional profile of the *cip-cel* operon in Fig. 1a.

Moreover, the top 2-GC-pair of IR10-SL are not perfect and its stem is much shorter than that of IR1-SL1 and IR5-SL1. To know whether the two 2-GC-pair regions of IR10-SL are important for RNA cleavage, they were replaced with UA-pairs (named IR10-SLΔGC1::UA and IR10-SLΔGC2::UA). The results of Northern blotting indicated that the replacement of the GC1 or GC2 region led to a significant reduction in the transcript level of monocistronic *fbfp* (Fig. 4i). It is consistent with the results in IR1-SL1 and IR5-SL1 confirming that two 2-GC-pair regions at the top and bottom of stem-loops played a crucial role in RNA cleavage.

To confirm the role of these two 2-GC-pair regions, they were respectively added to the top and bottom of a stem-loop (SLx) that is supposed not be cleaved, and the addition of 2-UA-pair regions were used as two control (Fig. 4j). As expected, the modified stem-loop RNA by GC-pairs acquired the ability to undergo cleavage that the control never did (Fig. 4k). Therefore, this result further demonstrated that the two 2-GC-pair regions located at the top and bottom of this stem-loop were crucial for RNA cleavage.

### Stems of Type II stem-loops required considerable length for RNA cleavage

**Fig. 5:**
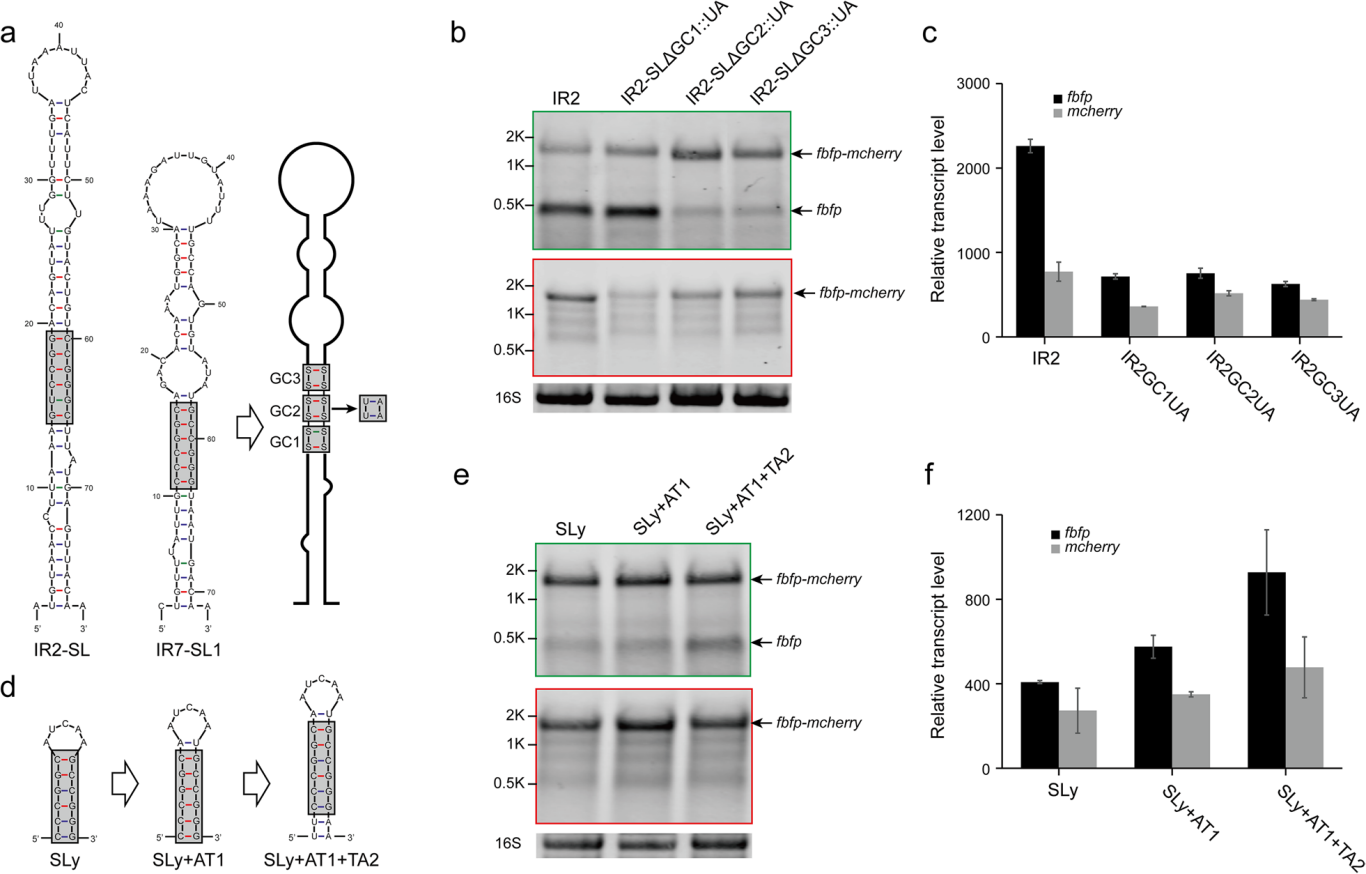
Structural requirement of Type II stem-loops for RNA cleavage. **a** Analysis of the structural conservation of Type II stem-loops located in IR2 and IR7, in which there is a 6-GC-pair region in their stems. **b**, **c** Effect of replacement of the 6-GC-pair region of IR2-SL with UA-pairs in the dual-fluorescence reporter system, as analyzed by Northern blotting (**b**) and qRT-PCR (**c**). **d**–**f** Stem-loop of Types II was designed (**d**) and its function was analyzed by Northern blotting (**e**) and qRT-PCR (**f**). Black arrows highlight the positions of bands that correspond to transcripts as indicated on the right side of the panel of Northern blotting results. 16S rRNA was used as a loading control. Error bars indicate the standard deviation of the mean from experiments done in triplicate

For Type II stem-loops from IR2 and IR7, a distinct 6-GC-pair region was located at their stems, although they harbored many unpaired regions (Fig. 5a). To identify their role in RNA cleavage, the 6-GC-pair region of the IR2 stem-loop was first substituted into UA-pairs two at a time (named IR2-SLΔGC1::UA, IR2-SLΔGC2::UA and IR2-SLΔGC3::UA, respectively). The results from Northern blotting showed that the RNA cleavage efficiency of IR2-SLΔGC2::UA and IR2-SLΔGC3::UA decreased dramatically, while that of IR2-SLΔGC1::UA was hardly changed (Fig. 5b). In contrast, the results of qRT-PCR indicated that the transcript levels of both *fbfp* and *mcherry* in derivatives of IR2 were much lower than that of wild-type IR2 transcripts (Fig. 5c). Thus, the results supported the contention that 6-GC-pair region of IR2 was important for RNA cleavage.

Furthermore, the 6-GC-pair region from IR7 was extracted to construct a simple stem-loop (SLy, Fig. 5d). Our results indicated that this new stem-loop had very low cleavage efficiency. However, its cleavage efficiency increased as its stem was lengthened, resulting in an increasing level of *fbfp* transcript (Fig. 5e, f). Therefore, although the 6-GC-pair region of stem-loops is important for RNA cleavage, their stems required a considerable length for efficient RNA cleavage to occur.

We thus hypothesized that there were two distinct types of stem-loops to be cleaved, one harbored two 2-GC-pair regions located at the top and bottom of a stem, and the other harboring a 6-GC-pair region in the middle of the stem.

### What determines the cleavage of IR4 occurring upstream of a stem-loop?

**Fig. 6:**
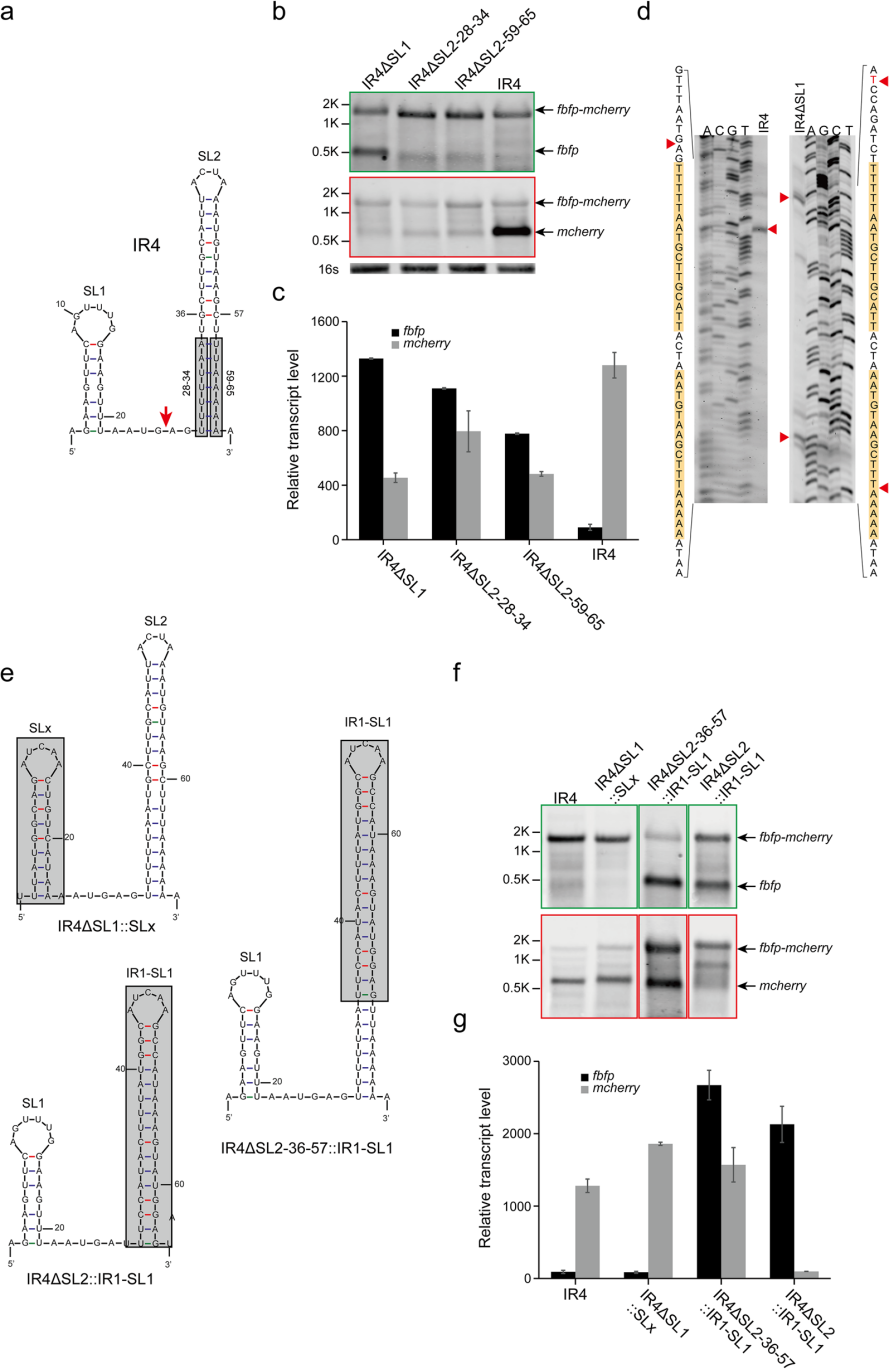
Mechanism of shifting RNA cleavage upstream of a stem-loop in IR4. **a** Secondary structure predicted for IR4 and a schematic diagram of mutation. The cleavage site is indicated by red arrows. **b**, **c** Effect of deletion of various elements of the secondary structure of IR4, as analyzed by Northern blotting (**b**) and qRT-PCR (**c**). **d** Cleavage sites of IR4 and IR4ΔSL1 were precisely identified by primer extension assay. The nucleotide sequences encompassing cleavage sites were listed on the right side of the panel. Cleavage sites are indicated by red arrows. (**e**) The schematic diagram of IR4 derivatives. **f**, **g** Effect of replacement of IR4-SL1 and IR4-SL2 with other stem-loops, as analyzed by Northern blotting (**f**) and qRT-PCR (**g**). Black arrows highlight the positions of bands that correspond to transcripts as indicated on the right side of the panel of Northern blotting results. 16S rRNA was used as a loading control. Error bars indicate the standard deviation of the mean from experiments done in triplicate

Unlike Type I and Type II stem-loops, which result in RNA cleavage downstream, the stem-loop of IR4 led to RNA cleavage upstream of the stem-loop. This stem-loop harbored an AT-pair region at its bottom, which was a characteristic that distinguished it from Type I and II stem-loops. Meanwhile, a small stem-loop (named SL1) was also predicted upstream of the cleavage site in IR4, in addition to the large stem-loop (named SL2) involved in RNA cleavage (Fig. 6a).

To identify the role that these elements played in RNA cleavage, several derivatives of IR4 were constructed. Surprisingly, the monocistronic transcripts of *fbfp* that were hardly detected in IR4 were detected decisively, and the abundance of monocistronic transcripts of *mcherry* was significantly decreased when SL1 in IR4 was deleted (IR4ΔSL1) (Fig. 6b), which was confirmed by the qRT-PCR results (Fig. 6c). The reason only monocistronic transcripts of *mcherry* were detected in IR4 was that the cleavage site was located upstream of SL2 resulting in processed *mcherry* harboring SL2 at its 5′ end. However, the opposite behavior was observed in IR4ΔSL1 in which the monocistronic transcripts of *fbfp* were greatly detected, suggesting that RNA cleavage in IR4ΔSL1 occurred downstream of SL2, resulting in processed *fbfp* harboring SL2 at its 3′ end. It was confirmed by the results of primer extension (Fig. 6d), in which another cleavage site was detected downstream of SL2 in IR4ΔSL1, in addition to the upstream cleavage site but it is positioned more forward than that of IR4. However, when SL1 was replaced with an artificial small stem-loop (SLx, Fig. 6e), the RNA cleavage in this IR4ΔSL1::SLx derivative was completely consistent with that of IR4 (Fig. 6f, g). Thus, the breakpoint of RNA cleavage in IR4 could be determined by SL1, but it was dependent on the structure of SL1 instead of its sequence.

Furthermore, both strands of the AT-pair region in SL2 were respectively deleted to generate two IR4 derivatives (named IR4ΔSL2-28-34 and IR4ΔSL2-59-65). The results from Northern blotting showed that the RNA cleavage efficiency of these two derivatives was decreased dramatically compared to IR4 (Fig. 6b). Meanwhile, the qRT-PCR results showed that the transcript level of *fbfp* was increased, while that of *mcherry* was decreased as the AT-pair region was abolished (Fig. 6c). Thus, the AT-pair region of SL2 also contributed to RNA cleavage of IR4. In contrast, the upper part of SL2 without the AT-pair region and the whole SL2 including the AT-pair region were replaced, respectively, with IR1-SL1, which could cause RNA cleavage downstream (Fig. 6e). We found that both processed monocistronic transcripts of *fbfp* and *mcherry* were present, as only the upper part of SL2 was replaced with IR1-SL1 (IR4ΔSL2-36-57::IR1-SL1), while only processed monocistronic transcripts of *fbfp* were detected when the intact SL2 was replaced (IR4ΔSL2::IR1-SL1) (Fig. 6f). This was consistent with our qRT-PCR results, in which the transcript level of *fbfp* from IR4ΔSL2-36-57::IR1-SL1 was dramatically increased and that of *mcherry* was almost as high as that for IR4. However, the transcript level of *fbfp* and *mcherry* in IR4ΔSL2::IR1-SL1 was the direct opposite of that in IR4 (Fig. 6g). These findings suggested that the AT-pair region could shift the cleavage site upstream of IR1-SL1 resulting in a high abundance of processed monocistronic *mcherry* transcripts, although IR1-SL1 alone only caused RNA cleavage at its downstream. Thus, the AT-pair region of SL2 not only affected the cleavage efficiency of IR4, but was also related to the breakpoint of RNA cleavage.

Therefore, these findings from IR4 revealed that SL1 and the AT-pair region of SL2 contributed together to the location of the cleavage site, while the sequences of SL1 and the upper part of SL2 seemed to be unimportant for RNA cleavage of IR4.

## Discussion

There has been a surge of new information about RNA processing in recent years. Nevertheless, more detailed information is required regarding the mechanisms responsible for RNA processing, such as the nature of cleavage sites, sequence specificity and the secondary structure context. In the present work, we explored the RNA processing sites and the cleavage signature of the *cip-cel* operon from *R. cellulolyticum*. The 12 genes of this operon encode critical cellulosomal components, including a primary scaffoldin subunit and cellulose-degrading enzymes, which dictate the capacity of this environmentally important, Gram-positive bacterium to carry out its primary function—the efficient degradation of cellulose.

### A potential endoribonuclease acts on *cip-cel* mRNA and its recognition sites

The main endoribonucleases that have been demonstrated to initiate RNA decay are RNase E in Gram-negative bacteria and RNase Y in Gram-positive bacteria [38, 39]. In addition, RNase J1/J2 also has been thought to be a contributor to mRNA degradation and turnover in Gram-positive bacteria [26, 40–42]. *R. cellulolyticum* harbors two typical Gram-positive bacterial endoribonucleases, RNase Y and RNase J, encoded by *Ccel_RS03075* (*rny*) and *Ccel_RS08950* (*rnj*), respectively. However, the gene *rny* encoding RNase Y could not be disrupted by clostron [43] despite our attempts, suggesting that it is essential for the growth of *R. cellulolyticum*, which was consistent with findings in other Gram-positive bacteria such as *B. subtilis* and *Clostridium perfringens* [44]. In contrast, *rnj* encoding RNase J can be disrupted by clostron but this does not affect the cleavage of *cip-cel* mRNA (Additional file 1: Fig. S4). Thus, we hypothesized that RNase Y was also the major initiator of primary *cip-cel* mRNA processing in *R. cellulolyticum*.

The additional requirements of RNase Y processing have been described in several Gram-positive bacteria. For example, the cleavage sites of RNase Y in *Streptococcus pyogenes* have been shown to exhibit a strong preference for a G residue based on RNA-seq data [18], while our results from primer extension assays showed that the cleavage sites of *cip-cel* transcripts seemed to be located in RNA single-stranded regions without preference (Additional file 1: Fig. S2). However, deletion of sequences encompassing the cleavage sites of the IRs did not prevent cleavage (Fig. 3), suggesting that RNase Y cleavage was not determined by the cleavage site.

Secondary structure prediction of IRs from the *cip-cel* operon revealed that endoribonucleases were likely to recognize stem-loop structures that include two types with distinct GC regions in their stems. Deletions of these stem-loops and mutations of their GC regions inhibited cleavage. These results indicated that the specificity for cleavage was determined by a stem-loop structure with specific stem sequences. The cleavage of the *yitY* leader in *B. subtilis* [45] and the *saePQRS* transcript in *S. aureus* [20] by RNase Y were also found to rely on RNA secondary structures, in which RNase Y recognizes a structure downstream of the cleavage site. However, in the *cip-cel* operon, we found that most of the cleavage sites were located downstream of recognized stem-loop structures, while their cleavage sites could be shifted upstream of the stem-loop structures when a small stem-loop and an AT-pair region were introduced, such as in IR4. Thus, the cleavage of IR4 makes its downstream processed transcript far more abundant than its upstream transcript due to protection of the stem-loop. In addition, if the stem-loop structure recognized by endoribonucleases is rich in U at its 3′ end such as the large stem-loop in IR5, it can also function as a rho-independent terminator.

### Stability of processed transcripts of the *cip-cel* operon

Our work revealed that the monocistronic transcripts of *cipC*, *celF* and *celE,* cleaved from the primary transcript of the *cip-cel* operon, harbored secondary structures at both their ends, resulting in the highest transcript levels among the *cip-cel* genes. The role of stem-loops at the ends of these processed transcripts was further investigated in our study. We found that addition or deletion of a stem-loop at both the 5′ and 3′ ends of processed transcripts increased or decreased the stability of these processed transcripts, suggesting that processed transcripts should be protected using secondary structures against both 3′-to-5′ exoribonucleases and 5′-to-3′ exoribonucleases in *R. cellulolyticum*. This could be explained by the observation that the 5′-to-3′ exoribonuclease RNase J (encoded by *Ccel_RS08950*) [9] was found to be present in *R. cellulolyticum*, similar to other Gram-positive bacteria, such as *B. subtilis*, in addition 3′-to-5′ exoribonucleases (PNPase [46] and RNase R [47], encoded by *Ccel_RS08610* and *Ccel_RS11355*, respectively)*.* In contrast, no processive RNA degradation activity in the 5′-to-3′ direction has been demonstrated in *E. coli* [48, 49]. The role of RNase J in the *cip-cel* mRNA was further confirmed by the *rnj* mutant, in which the transcript level of the *cip-cel* operon was much higher than wild-type, suggesting that RNase J contributes to the stability of the *cip-cel* mRNA (Additional file 1: Fig. S4).

Furthermore, the structure of the stem-loops at the 3′ end of processed transcripts, such as IR1-SL1, IR2-SL, IR5-SL1, IR7-SL1 and IR10-SL, is more complex than those at the 5′ end, including IR1-SL2, IR4-SL, IR5-SL2, and IR7-SL2, that is consistent with our previous observations of stronger protection of the 3′ end in *R. cellulolyticum* [9, 50]. Together, the structural difference of stem-loops at both ends of processed transcripts suggested that the activity of 3′-to-5′ exoribonucleases was much higher than that of 5′-to-3′ exoribonucleases in *R. cellulolyticum*, resulting in stronger protection at the 3′ end of processed transcripts.

Thus, the fate of processed transcripts after cleavage by RNase Y could be determined by the secondary structures at their ends which involved in protection and RNA fragments stabilization, the processing fragments were highly resistant to degradation because they were protected by this structure. From our data, we conclude that two different consequences were conceivable: processed transcripts harboring secondary structures at their ends become more stable, resulting in higher abundance; or, conversely, they become more unstable and have lower abundance.

### An updated model for the regulation of the stoichiometry of the *cip-cel* operon

In this work, we determined that RNA cleavage events happen in the IRs of the *cip-cel* operon and elucidated their potential recognition determinants by endoribonucleases in *R. cellulolyticum* through the artificial bicistronic operons. These new findings allowed us to improve and correct our previous model for regulation of the stoichiometry of cellulosomal components from the *cip-cel* operon, as proposed using transcriptomic data [9]. First, two new cleavage events in IR7 and IR10 were found in this study, in addition to the first four that were found in a previous work. However, they can be further demonstrated in vivo to occur on *cip-cel* operon due to low abundance of their natural transcripts. Second, it was previously surmised that all cleavage sites were located downstream of the major stem-loops. However, it was clearly demonstrated in the present work that the cleavage in IR4 is an exception. Third, the major stem-loop in IR5 has another function in addition to RNA cleavage. It could prematurely terminate the transcription of the *cip-cel* operon as an internal rho-independent terminator (Fig. 4). Finally, processed transcripts with high abundance, such as *cel48F* and *cel9E*, harbored stem-loop structures at both the 5′ and 3′ ends, and not only at their 3′ ends as previously thought.

**Fig. 7:**
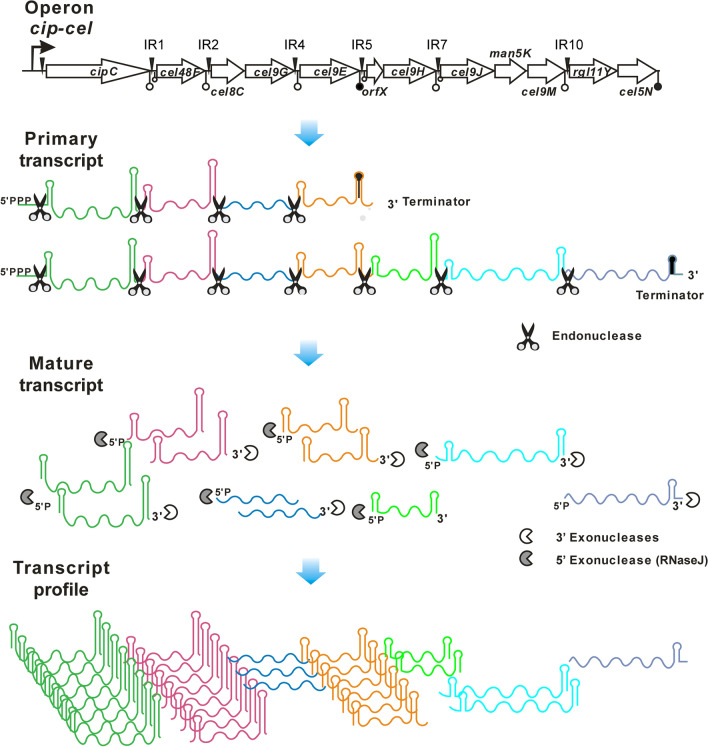
Updated model for the regulation of the stoichiometry of cellulosomal components in vivo. The *cip-cel* operon is transcribed by its promoter into the primary *cip-cel* mRNA, but most transcripts are prematurely terminated by an internal terminator at IR5. Cleavage signals located in the six intergenic regions are specifically recognized by endoribonucleases, resulting in cleavage of the primary transcript into several secondary transcripts. Stability of these secondary transcripts varies widely due to their distinct structures at both ends, which convey resistance to exoribonuclease degradation

Therefore, the *cip-cel* operon is transcribed by its promoter into the primary *cip-cel* mRNA, but most transcripts can be prematurely terminated by an internal terminator at IR5. Cleavage signals in the six IRs proved by the artificial bicistronic operons are specifically recognized by endoribonucleases, resulting in cleavage of the primary transcripts. The stability of these secondary transcripts varies widely due to their distinct structures at both ends, which convey resistance to exoribonuclease degradation. The processed transcripts of *cipC*, *cel48F*, and *cel9E* appear to be protected by stem-loops at both ends, leading to their significant abundance. However, although the processed transcripts of *orfX*-*cel9H* and *cel9J*-*cel9M* also harbor stem-loops at both ends, their abundance is still lower than that of processed transcripts of *cel8C*-*cel9G*, which lack any stem-loops at their ends, because they are located downstream of the internal transcription terminator (Fig. 7). Therefore, the specific stoichiometry of cellulosomal subunits encoded in the *cip-cel* operon match well with their functions. For example, the most highly-expressed GH48 exocellulase and endo-processive GH9 cellulase encoded by *cel48F* and *cel9E* are crucial to hydrolysis of crystalline cellulose [51].

The key regulators discovered here, such as the stem-loops, whose strength for promoting transcript abundance is encoded by the primary sequence alone, imply the existence of an efficient approach to specify and tune the relative transcript levels of genes within a single operon.

## Conclusion

In this work, cleavage events in the *cip-cel* operon of *R. cellulolyticum* were investigated and found to occur at six IRs harboring stem-loop structures. The analyses of processed transcripts validated that all cleavages of the *cip-cel* mRNA were determined by their nearby stem-loops, which included two distinct types of stem sequences which are specifically recognized by endoribonucleases. Cleavage sites have been demonstrated that are located downstream of their associated stem-loops, whereas the cleavage site in IR4 is located upstream of the stem-loop, as determined by the bottom AT-pair region of the stem-loop together with its upstream structure. Remarkably, the stem-loop structures that lie in the processed mRNA 5′/3′ ends could protect processed transcripts against exoribonucleases. This study updates the model for regulation of the stoichiometry of the *cip-cel* operon and unveils the sequence/structure requirements for processing of the *cip-cel* transcripts, which has general implications in designing synthetic elements to control expression stoichiometry of genes in an operon.

## Materials and methods

### Strains and culture conditions

The bacterial strains and plasmids used in this study are listed in Additional file 1: Table S1. *Escherichia coli* DH5α was used as the host strain for the recombinant plasmids constructions and was grown at 37 °C in Luria–Bertani (LB) medium. *R. cellulolyticum* ATCC35319 (H10) was cultured anaerobically at 35 °C in GS-2 medium supplemented with 3 g L^−1^ of cellobiose as the sole carbon source [52]. The cultures were inoculated with exponentially growing cells and harvested in the exponential growth phase after reaching an OD_600_ of 0.8–1.0. When required, antibiotics were added at the following concentrations: 100 μg mL^−1^ ampicillin or 20 μg mL^−1^ erythromycin.

### Plasmid construction and transformation

All plasmids constructed in this study are listed in Additional file 1: Table S1. IRs and its derivatives were respectively fused with the *mcherry* gene (encoding a red fluorescence protein) by overlapping extension (SOE) PCR using synthetic oligonucleotide primers with *R. cellulolyticum* genomic DNA used as a template. The fused fragments were subsequently digested with BglII and EcoRI and cloned between the corresponding site of pMCT6 [9]. In the resultant plasmids, IRs were inserted between *fbfp* and *mcherry* and they were expressed in a single operon, driven by the *Pthl* promoter (the thiolase gene promoter from *Clostridium acetobutylicum*) [53]. *R. cellulolyticum* cells were electro-transformed anaerobically with all plasmids according to the literature protocol [52] and transformants were isolated on selective solid medium (GS-2) containing 20 μg mL^−1^ erythromycin. All primers used are listed in Additional file 1: Table S2.

### Analysis of mutant strains

pSY6-RS08950 for targeted disruption of *R. cellulolyticum* gene *Ccel_RS08950* (encoding nuclease RNase J) was constructed from clostron plasmid pSY6 [54]. The targeting site for disruption and the clostron-targeting primers were designed with tools based on the Perutka algorithm (http://clostron.com/) [43]. The clostron-targeting region, which was obtained by SOE-PCR with the primer shown in Additional file 1: Table S2, was inserted into pSY6 after digestion with XhoI and BsrGI for targeted disruption of *Ccel_RS08950*.

### Northern blotting

Total RNA was isolated from *R. cellulolyticum* cultures on cellobiose using an EZ-10 Total RNA Mini-Prep Kit (Sangon, China). Five micrograms of RNA samples were separated via electrophoresis on 1% denaturing gel agarose gel (10× FA buffer, 0.2% formaldehyde) and blotted onto a N+ nylon membrane (Hybond-NX, GE HealthCare, USA) using the Northern MaxTM-Gly kit Complete Northern Blotting (ThermoFisher, USA). Cy5.5-labelled DNA probes for the detection of *fbfp* and *mcherry* were directly prepared commercially (Sangon, China) using the oligonucleotides in which Cy5.5 was coupled to the 5′ end of the oligonucleotides as listed in Additional file 1: Table S2. The signals were detected with an Odyssey CLx dual-color infrared laser imaging system (LI-COR, USA) at 720 nm. Steps of Northern blotting were detailed in our previous study [55]. 16s rRNA stained with methylene blue on the blots is indicated at the bottom as a loading control.

### Primer extension

The processing site was mapped by reverse transcription of the processed transcript using an oligonucleotide (showed in Additional file 1: Table S2) complementary to positions 16–25 bp of the *mcherry*, a sequencing ladder starting at the 5′end of the transcript was run in parallel. In the case of the full-length transcript, the second fragment originating from this cleavage site has a predicted length about 150 bp corresponding to the sequences between the cleavage site and the 3′end of the transcript.

To map the 5′-terminal nucleotide of processed mRNA, the mixture of total RNA (10 μg) and the Cy5.5-labeled oligonucleotide primer were denatured 5 min at 65 °C and subsequently incubated on ice for 2 min. Samples were reverse transcribed using 200 U of Hifair IV reverse transcriptase (Yeasen, China) in the presence of 5× first strand buffer, 5 mM dNTP Mix and 40 U of Recombinant Ribonuclease Inhibitor (Takara, Japan), as per the manufacturer’s instructions. Thermal cycling conditions were as follows: 25 °C for 5 min, 55 °C for 15 min and 85 °C for 5 min. The cDNA products were analyzed on an 8% polyacrylamide sequencing gel (8 M urea/TBE). Sequencing ladders were generated using USB Thermo Sequenase Cycle Sequencing Kit (Thermo Fisher Scientific, USA) with the same Cy5.5-labeled oligonucleotide primer, and the recombinant plasmids digested by BglII is as template.

### Quantitative reverse transcription-PCR (qRT-PCR)

To analyze the effect of the introduction of IRs and their derivatives to transcription of the upstream and downstream genes in the dual-fluorescence reporter system, the relative transcript level of *fbfp* and *mcherry* was measured via qRT-PCR. The cDNA product was obtained by qRT-PCR using the Hiscript ® III RT SuperMix for qRT-PCR (+gDNA wiper) kit as described by the manufacturer’s instructions. The qRT-PCR was performed using ChamQ Univeral SYBR qPCR Master Mix (Vazyme Biotech, China) on CFX96 real-time PCR detection system (Bio-Rad, USA) and the results normalized via abundance of *Ccel_RS01560* encoding the β subunit of DNA-directed RNA polymerase. The primer sets for qRT-PCR are listed in Additional file 1: Table S2.

### mRNA stability assays

For mRNA stability assays, 80 μg mL^−1^ of rifampicin (Sangan, China) was added to exponentially growing *R. cellulolytiucm* cells to prevent transcription initiation. Samples for RNA preparation were removed before and at different times (at 0, 5, 10, and 15 min) after rifampin addition, and preparation of total RNA was carried out as described previously [15]. Northern blotting was then performed as described above to determine the relative level of *fbfp* or *mcherry* expression for each mutation at the given time points after rifampin addition.

### RNA secondary structure prediction

The sequences of the intergenic regions (IRs) are exactly from the first nucleotide after stop codon of the upstream gene to the last nucleotide before start codon of the downstream gene according to the genome annotation of *R. cellulolyticum* in NCBI (ID: NC_011898). The full RNA secondary structure of the sequences as defined above was computed using Mfold with default parameters (http://unafold.rna.albany.edu/). The output secondary structures were collected and analyzed for the presence of stems and loops.

## Supplementary Information

**Additional file 1: Figure S1.** Secondary structure prediction of all intergenic regions (IRs) from *cip-cel* operon. **Figure S2.** RNA cleavage sites were precisely identified by primer extension assay. **Figure S3.** Analysis of the stability of *fbfp* mRNA. **Figure S4.** The effect of RNase J on RNA cleavage of the *cip-cel* operon. **Table S1.** Strains and plasmids used in this study. **Table S2.** Primers used in this study. **Table S3.** Sequences of IRs in the *cip-cel* operon.

## Data Availability

All data are available in the main text or Additional file.
