# Supplementary material for: Stem-loop structures control mRNA processing of the cellulosomal cip-cel operon in Ruminiclostridium cellulolyticum
Source: Biotechnol Biofuels Bioprod. 2023 Jun 29;16:106. doi: 10.1186/s13068-023-02357-5 (PMC10311766; doi:10.1186/s13068-023-02357-5)
Supplement: Supplementary file 1 — Additional file 1: Figure S1. Secondary structure prediction of all intergenic regions (IRs) from cip-cel operon. Figure S2. RNA cleavage sites were precisely identified by primer extension assay. Figure S3. Analysis of the stability of fbfp mRNA. Figure S4. The effect of RNase J on RNA cleavage of the cip-cel operon. Table S1. Strains and plasmids used in this study. Table S2. Primers used in this study. Table S3. Sequences of IRs in the cip-cel operon. [file 13068_2023_2357_MOESM1_ESM.docx]

**Additional file 1**

**Additional Figures:**

**
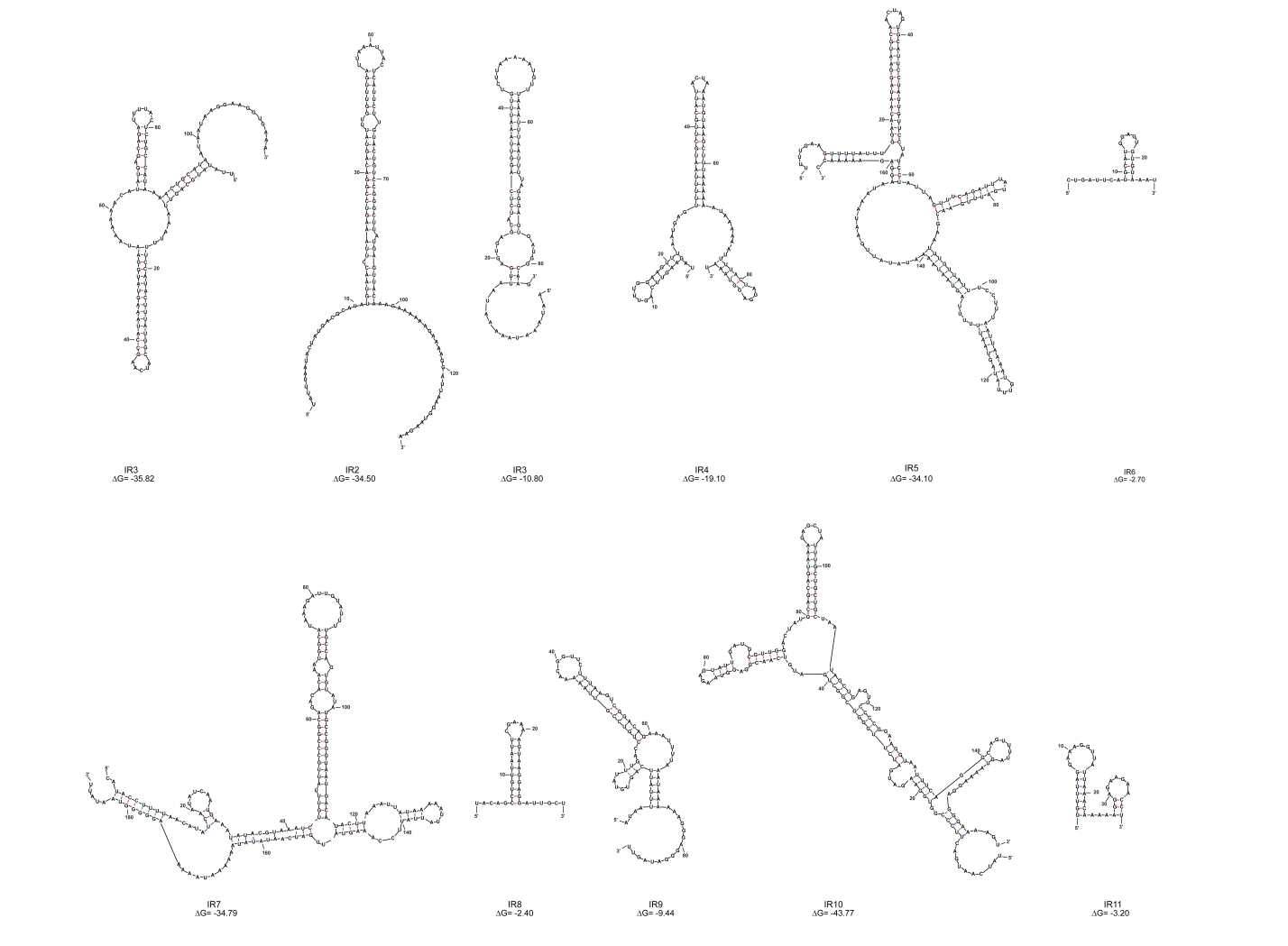
**

**Fig. S1. Secondary structure prediction of all intergenic regions (IRs) from *cip-cel* operon.**


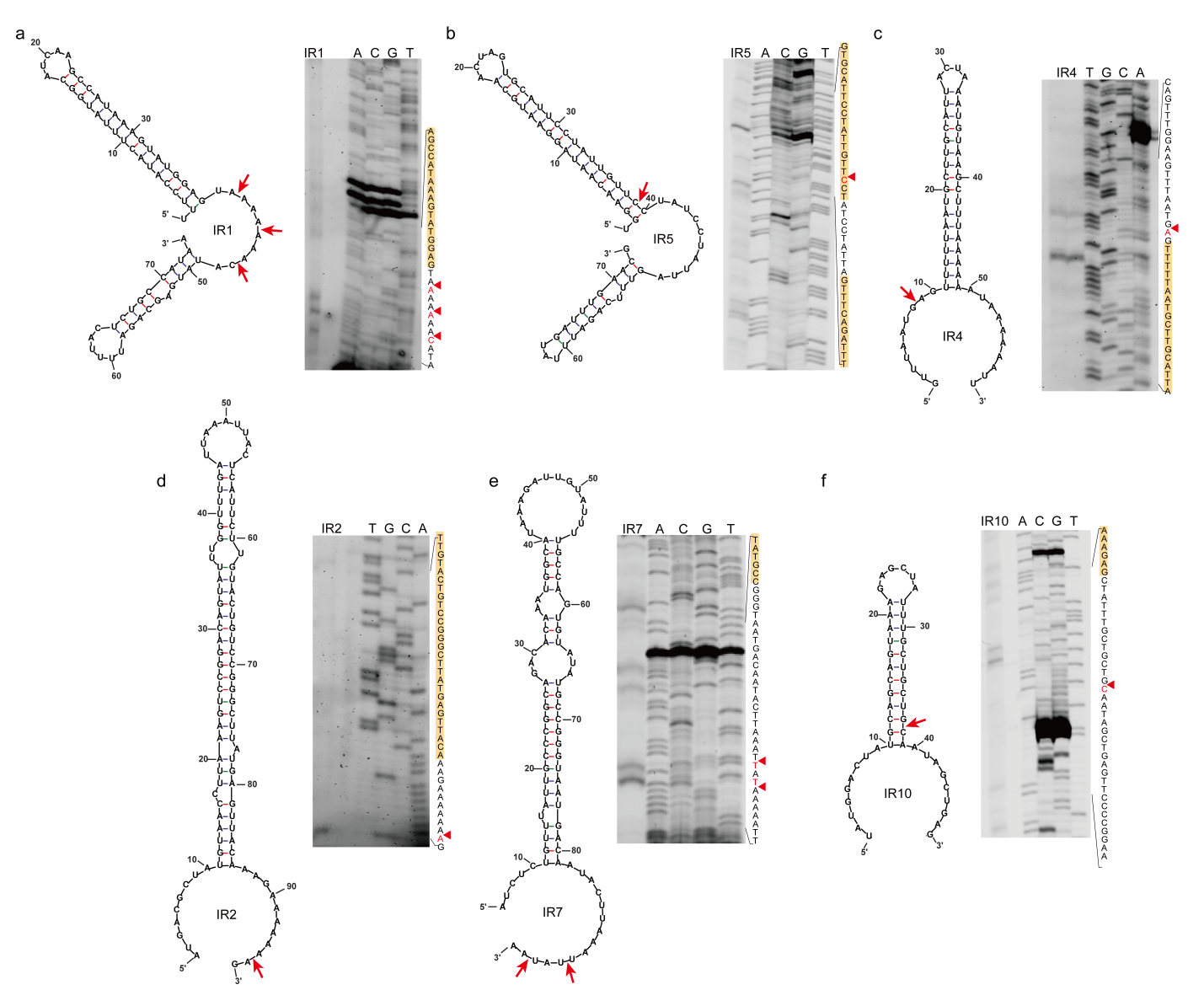


**Fig. S2. RNA cleavage sites were precisely identified by primer extension assay.** Reverse transcription was performed as described in the Materials and Methods with a Cy5.5-labelled oligonucleotide (Mcherry-P1), which starts at 25 bp downstream of the initiation codon of *mcherry*. The terminated and full-length transcripts were separated on a 8% polyacrylamide gel. The nucleotide sequences encompassing cleavage sites were listed on the right side of the panel. Cleavage sites of IR1 (**a**), IR5 (**b**), IR4 (**c**), IR2 (**d**), IR7 (**e**), and IR10 (f) are respectively indicated by red arrows.


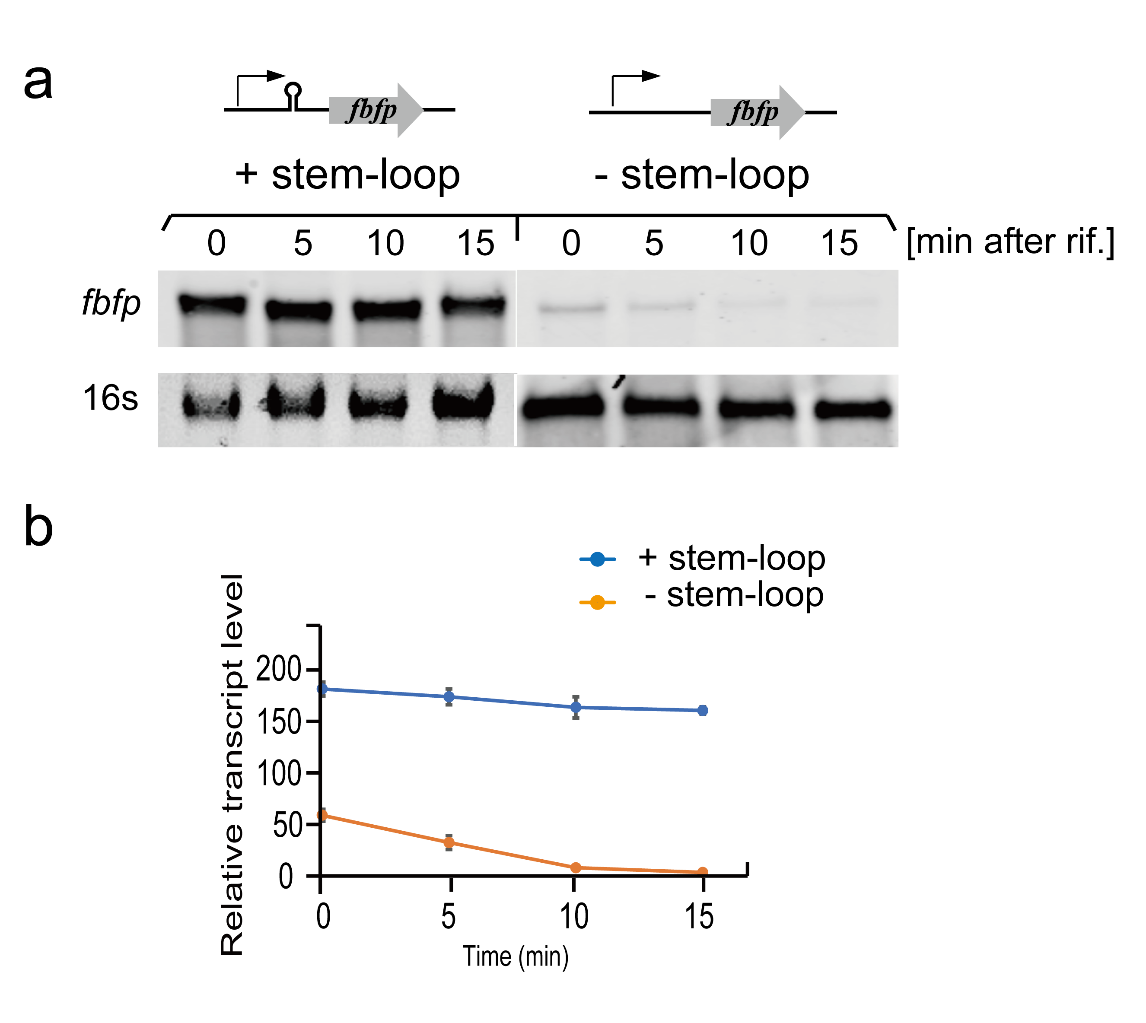


**Fig. S3. Analysis of the stability of *fbfp* mRNA.** (**a**) Northern blotting analysis of transcripts of *fbfp* with or without a stem-loop at its 5’ end at intervals after rifampin addition. 16S rRNA was used as a loading control. (**b**) Half-lives of the *fbfp* transcript (+stem-loop:45.3min, -stem-loop:5.3 min) were calculated by comparison of the transcript abundance of *fbfp*. Error bars indicate the standard deviation of the mean from experiments done in triplicate.


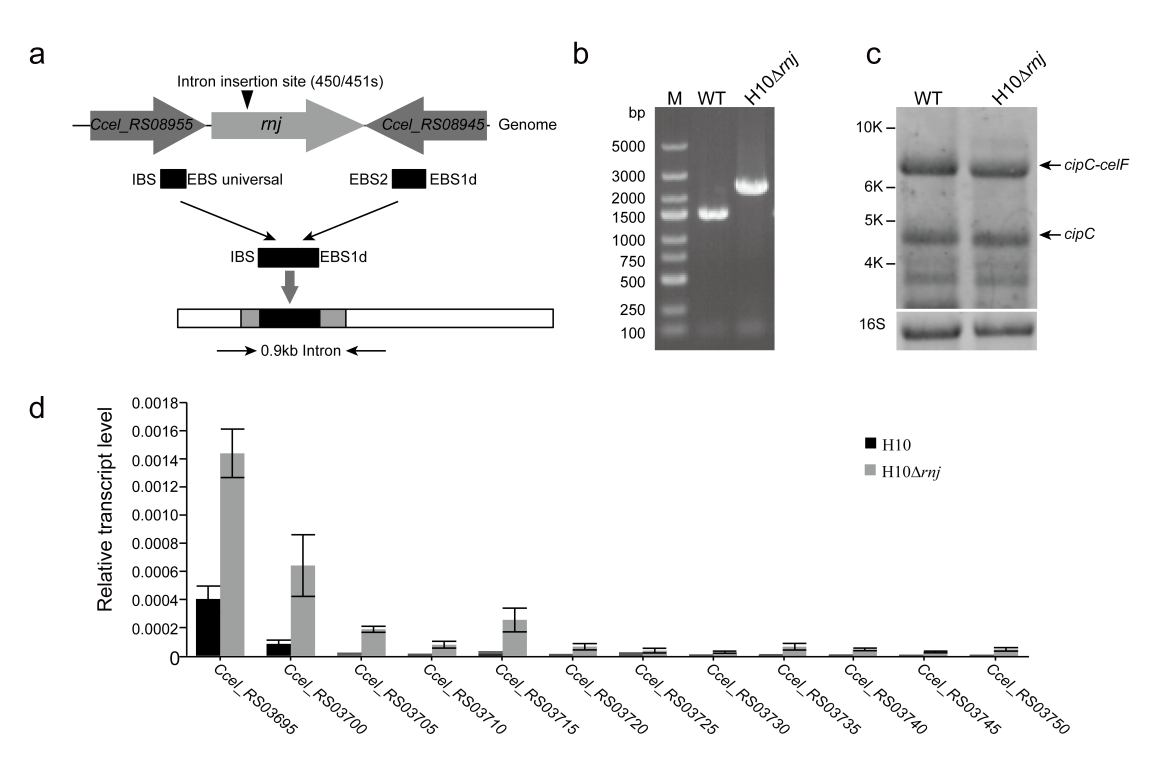


**Fig. S4. The effect of RNase J on RNA cleavage of the *cip-cel* operon.** (**a**) Gene loci of *rnj* encoding RNase J in *R. cellulolyticum* H10. The inserted site of clostron for disruption was indicated. (**b**) PCR confirmation that *rnj* is disrupted by clostron resulting a 0.9-kb insertion. (**c**) Transcription of *cipC* between wild type (WT) and *rnj*-disrupted mutant of *R. cellulolytiucm* H10 (H10Δ*rnj*) was analyzed by Northern blotting using the probe targeting *cipC*. 16S rRNA was used as a loading control. (d)The stability of eleven genes of the *cip-cel* operon were detected by qRT-PCR in WT and the *rnj* mutant. Data were normalized via the transcript level of the gene of *Ccel_RS01560*, encoding the RNA polymerase beta subunit. Error bars indicate the standard deviation of the mean from experiments done in triplicate.

**Additional Tables:**

**Table S1. Strains and plasmids used in this study**

| Strains and plasmids | Relevant characteristic(s) | Source or references |  |
| --- | --- | --- | --- |
| **Strains** |  |  |  |
| *E. coli* DH5α | *f80dlacZΔM*15*, Δ(lacZYA-argF)U*169*, endA*1*, recA*1 *hsdR*17*(r_k_^-^, m_k_^+^), supE*44*, l^-^ thi-1 gyrA*96, *relA*1*, phoA* | Transgene |  |
| *R.cellulolytiucm* H10∆*mspI* | Derived from *Ruminiclostridium cellulolytiucm* H10 with the *mspI* deleted | Granted from Cui *et al* ^a^ |  |
| *R.cellulolytiucm* H10∆*mspI*∆*rnj* | Derived from *Ruminiclostridium cellulolytiucm* H10∆*mspI* with *rnj* (*Ccel_RS08950*) deleted | This study |  |
| **Plasmids** | | |  |
| pMTC6 | Derived from pIMPI, containing *PpFbFpm, thl* promoter | Granted from Cui *et al* ^a^ | |
| pSY6 | MlsR, AmpR, *E. coli–R. cellulolyticum* shuttle vector, *ptb* promoter, containing *L. lactis* L1. Ltr intron and *ltrA* | Shao et.al ^b^ | |
| pSY6-RS08950 | Derived from pSY6, targeting the *Ccel_RS08950* in *R. cellulolyticum* | This study | |
| pfbfp+SL | pMTC6 derivative expression vector harboring IR1-SL2 | This study | |
| pIR1 | pMTC6 derivative expression vector harboring IR1 | This study | |
| pIR2 | pMTC6 derivative expression vector harboring IR2 | This study | |
| pIR3 | pMTC6 derivative expression vector harboring IR3 | This study | |
| pIR4 | pMTC6 derivative expression vector harboring IR4 | This study | |
| pIR5 | pMTC6 derivative expression vector harboring IR5 | This study | |
| pIR6 | pMTC6 derivative expression vector harboring IR6 | This study | |
| pIR7 | pMTC6 derivative expression vector harboring IR7 | This study | |
| pIR8 | pMTC6 derivative expression vector harboring IR8 | This study | |
| pIR9 | pMTC6 derivative expression vector harboring IR9 | This study | |
| pIR10 | pMTC6 derivative expression vector harboring IR10 | This study | |
| pIR11 | pMTC6 derivative expression vector harboring IR11 | This study | |
| pSLx | pMTC6 derivative expression vector harboring SLx | This study | |
| pSLy | pMTC6 derivative expression vector harboring SLy | This study | |
| pIR1∆SL1 | pIR1 derivative with SL1 deleted | This study | |
| pIR1∆linker | pIR1 derivative with Linker deleted | This study | |
| pIR1∆SL2 | pIR1 derivative with SL2 deleted | This study | |
| pIR2∆stem | pIR2 derivative with stem regions deleted | This study | |
| pIR2∆linker | pIR2 derivative with Linker deleted | This study | |
| pIR2∆Loop | pIR2 derivative with loop of SL deleted | This study | |
| pIR2∆Bubble | pIR2 derivative with bubble of stem deleted | This study | |
| pIR2::SL2 | pIR2 derivative with IR1-SL2 added into the downstream of IR2 | This study | |
| pIR4∆stem | pIR4 derivative with stem regions deleted | This study | |
| pIR4∆SL1 | pIR4 derivative with SL1 deleted | This study | |
| pIR4::SL2 | pIR4 derivative with IR1-SL2 added into the upstream of IR4 | This study | |
| pIR5∆SL1 | pIR5 derivative with SL1 deleted | This study | |
| pIR5∆Linker | pIR5 derivative with Linker deleted | This study | |
| pIR5∆SL2 | pIR5 derivative with SL2 deleted | This study | |
| pIR7∆SL1 | pIR7 derivative with SL1 deleted | This study | |
| pIR7∆Linker | pIR7 derivative with Linker deleted | This study | |
| pIR7∆SL2 | pIR7 derivative with SL2 deleted | This study | |
| pIR7∆stem | pIR7 derivative with stem regions deleted | This study | |
| pIR7∆Loop | pIR7 derivative with loop of SL deleted | This study | |
| pIR7∆Bubble | pIR7 derivative with bubble of stem deleted | This study | |
| pIR10∆stem | pIR10 derivative with stem regions deleted | This study | |
| pIR1-SL1∆GC1 | pIR1 derivative with GC1 of SL1 deleted | This study | |
| pIR1-SL1∆GC2 | pIR1 derivative with GC2 of SL1 deleted | This study | |
| pIR1-SL1∆GC1∆GC2 | pIR1 derivative with both GC1 and GC2 of SL1 deleted | This study | |
| pIR1-SL1  ΔCCGG::GGCC | pIR1 derivative with the order of GC1 (CCGG) mutated into GGCC | This study | |
| pIR1- SL1  ΔCCGG::CGCG | pIR1 derivative with the order of GC1 (CCGG) mutated into CGCG | This study | |
| pIR1- SL1  ΔCCGG::GCGC | pIR1 derivative with the order of GC1 (CCGG) mutated into GCGC | This study | |
| pIR5-SL1ΔGC1 | pIR5 derivative with GC1 of SL1 deleted | This study | |
| pIR5-SL1ΔGC2 | pIR5 derivative with GC2 of SL1 deleted | This study | |
| pIR5-SL1ΔGC2ΔGC3 | pIR5 derivative with both GC2 and GC3 of SL1 deleted | This study | |
| pIR5-SL1ΔGC3 | pIR5 derivative with GC3 of SL1 deleted | This study | |
| pIR2-SLΔGC1::UA | pIR2 derivative with the GC1 of stem mutated into UA-pair | This study | |
| pIR2-SLΔGC2::UA | pIR2 derivative with the GC2 of stem mutated into UA-pair | This study | |
| pIR2-SLΔGC3::UA | pIR2 derivative with the GC3 of stem mutated into UA-pair | This study | |
| pIR4ΔSL1::SLx | pIR4 derivative with the SL1 replaced by SLx | This study | |
| pIR4ΔSL2-28-34 | pIR4 derivative with 5’ single strand of AT-pair region (28-34) of SL2 deleted | This study | |
| pIR4ΔSL2-59-65 | pIR4 derivative with 3’ single strand of AT-pair region (59-65) of SL2 deleted | This study | |
| pIR4ΔSL2-36-57::IR1  -SL1 | pIR4 derivative with the upper part of SL2 replaced with IR1-SL1 | This study | |
| pIR4ΔSL2::IR1-SL1 | pIR4 derivative with SL2 were replaced with IR1-SL1 | This study | |
| pIR10-SLΔGC1::UA | pIR10 derivative with the GC1 of stem mutated into UA-pair | This study | |
| pIR10-SLΔGC2::UA | pIR10 derivative with the GC2 of stem mutated into UA-pair | This study | |
| pSLx+GC1 | pSLx derivative with GC1 added into the bottom of stem | This study | |
| pSLx+GC1+GC2 | pSLx derivative with GC1 and GC2 added into the bottom and top of stem respectively | This study | |
| pSLx+UA1 | pSLx derivative with UA1 added into the bottom of stem | This study | |
| pSLx+UA1+UA2 | pSLx derivative with UA1 and UA2 added into the bottom and top of stem respectively | This study | |
| pSLy+AT1 | pSLy derivative with AU1 added into the top of stem | This study | |
| pSLy+AT1+TA2 | pSLy derivative with both AU1 and UA2 added into the top and bottom of stem | This study | |

^a^ Cui, Hong et al. 2012

^b^ Shao, Hu et al. 2007

Table S2. Primers used in this study

| primers | Sequence (5’-3’) | | Description |
| --- | --- | --- | --- |
| For cloning of intergenic regions (IRs) | | | |
| IR1_F | | GGAAGATCTTTATTGAATTTAAATTTTCCATAC | To amplify the intergenic regions of *cipC*-*cel48F* (IR1) |
| IR1_R | | CTTCTCCTTTTGATACCATTTTACACCTTCCTTATTCTTTGAA |  |
| IR2_F | | GGAAGATCTTATTTAATACTATGACGCATATG | To amplify the intergenic regions of *cel48F*-*cel8C* (IR2) |
| IR2_R | | CTTCTCCTTTTGATACCATGTTCTTACCTTAATCCTTTTC |  |
| IR3_F | | GGAAGATCTAATAAATAAAAATAATTGAGTGAG | To amplify the intergenic regions of *cel8C*-*cel9G* (IR3) |
| IR3_R | | CTTCTCCTTTTGATACCATCTTGCCATCACTCCCTAAAATT |  |
| IR4_F | | GGAAGATCTTAGAAGTTCAGTTTGGAAGTT | To amplify the intergenic regions of *cel9G*-*cel9E* (IR4) |
| IR4_R | | CTTCTCCTTTTGATACCATATTTACCTCCTAGTAAAATTTTTTAT |  |
| IR5_F | | GGAAGATCTTTTTGAAGTTTTATTTGGAAC | To amplify the intergenic regions of *cel9E*-*orfX* (IR5) |
| IR5_R | | CTTCTCCTTTTGATACCATGGCTTTTCTCCTTATTTATTCAA |  |
| IR6_F | | GGAAGATCTGAACTCAAGAAGATTAATCCT | To amplify the intergenic regions of *orfX*-*cel9H* (IR6) |
| IR6_R | | CTTCTCCTTTTGATACCATATTTACACACTCCATGCATGAATCAG |  |
| IR7_F | | GGAAGATCTCATACCTTTTAACATATCAAT | To amplify the intergenic regions of *cel9H*-*cel9J* (IR7) |
| IR7_R | | CTTCTCCTTTTGATACCATAATATTACCCCCTTTTTATTTTTTATATATTG |  |
| IR8_F | | GGAAGATCTTGCTCTTGACCTTGCTTTATTA | To amplify the intergenic regions of *cel9J* -*Man5K* (IR8) |
| IR8_R | | CTTCTCCTTTTGATACCATAGCAAATCCTCCTTACTTTTCGAATTAAC |  |
| IR9_F | | GGAAGATCTATAATTTATTACAGTGTATTTGAC | To amplify the intergenic regions of *Man5K*-*cel9M* (IR9) |
| IR9_R | | CTTCTCCTTTTGATACCATAACTATCCCTCCCTTTTTTATTTAAAAATTTCTGTCCG |  |
| IR10_F | | GGAAGATCTTATCAATGACTTCCCGGTGGA | To amplify the intergenic regions of *cel9M* -*rgl11Y* (IR10) |
| IR10_R | | CTTCTCCTTTTGATACCATACTTTTCCCTCCTTTTACTAAAACTGCC |  |
| IR11_F | | GGAAGATCTCACTAGACTTGGCATTAC | To amplify the intergenic regions of *rgl11Y* -*cel5N* (IR11) |
| IR11_R | | CTTCTCCTTTTGATACCATAGGTTCTTCTCCTTTTTGTTTAATAACC |  |
| For deleting the specific regions of IRs | | | |
| IR1∆SL1_F | | GGAAGATCTTTATTGAATTTAAATTTAAAAAAACATATGAGCAG | To amplify IR1 without SL1 |
| IR1∆Linker_F1 | | GGCATCAAGCCATAAAGTATGGAGTATGAGCAGATTTTACTC | To amplify IR1 without Linker between stem-loops |
| IR1∆Linker_F2 | | TTTCCATACTTTATGGCATCAAGCCATAAAGTATG |  |
| IR1∆Linker_F3 | | GGAAGATCTTTATTGAATTTAAATTTTCCATACTTTATGGCATCA |  |
| IR1∆SL2_F1 | | CATAAAGTATGGAGTAAAAAAACAAAAATTCAAAGAATAAGG | To amplify IR1 without SL2 |
| IR1∆SL2_F2 | | CCATACTTTATGGCATCAAGCCATAAAGTATGGAGTAAAAAAAC |  |
| IR1∆SL2_F3 | | GGAAGATCTTTATTGAATTTAAATTTTCCATACTTTATGGCATCAAGC |  |
| IR2∆Linker_F | | GGAAGATCTTGTAACCTTAAAGTCCGGACAG | To amplify IR2 without Linker between stem-loops |
| IR2∆stem_F | | AATACTATGACGCATATGTAACCTTATTAAATTACTGAGTTACAAAGAAAAAAAG | To amplify IR2 without stem regions of stem-loop |
| IR2∆Loop_F1 | | GACAGTATTTAAATTACGTACTGTCCGGGCTTATGAG | To amplify IR2 without Loop of stem-loop |
| IR2∆Loop_F2 | | ATACTATGACGCATATGTAACCTTAAAGTCCGGACAGTATTTAAATTAC |  |
| IR2∆Bubble_F | | GGAAGATCTTATTTAATACTATGACGCATATGTAACTTAAAGTCCGG | To amplify IR2 without Bubble of stem-loop |
| IR2∆Bubble_R | | GTTCTTACCTTAATCCTTTTCTTTTTTTCTTTGTAACTCAAAGCCC |  |
| IR4∆stem_F1 | | GTTTAATGAGTTGCATTACTAAATGTAAATAAAAAATTTTACTAGGAGG | To amplify IR4 without stem regions of stem-loop |
| IR4∆stem_F2 | | GGAAGATCTTAGAAGTTCAGTTTGGAAGTTTAATGAGTTGCATTACTAAATG |  |
| IR5∆SL1_F | | GGAAGATCTTTTTGAAGTTTTATTTTATCCTATTAGTTTCAGATTTATG | To amplify IR5 without SL1 |
| IR5∆SL2_F1 | | GGAATGCAACTAGTGCATTCCTATTGTTCCTATCCTATTAGAATTTGTTATTTCC | To amplify IR5 without SL2 |
| IR5∆SL2_F1 | | GGAAGATCTTTTTGAAGTTTTATTTGGAACAATAGGAATGCAACTAGTGCATTC |  |
| IR5∆Linker_F1 | | GGAATGCAACTAGTGCATTCCTATTGTTCCGTTTCAGATTTATGATTTGAACG | To amplify IR5 without Linker between stem-loops |
| IR5∆Linker_F2 | | GGAAGATCTTTTTGAAGTTTTATTTGGAACAATAGGAATGCAACTAGTGCATTCC |  |
| IR7∆SL1_F1 | | CAATGAAATATACGTAAATCTCATACTTAAATTATAAAAATTATT | To amplify IR7 without SL1 |
| IR7∆SL1_F2 | | GGAAGATCCATACCTTTTAACATATCAATATCAATGAAATATACGTAAATCTCATAC |  |
| IR7∆SL2_F | | GGAAGATCCATACCTTTTAACATATCAATATC | To amplify IR7 without SL2 |
| IR7∆SL2_R | | CTTCTTCTCCTTTTGATACCATAATATTACCCCCTTTTTATTTTTTATATATTGATCTTTATAATTT |  |
| IR7∆Linker_R1 | | ATATATTGATCAACTACTTTGGAATAATAATTTGTCATTACCCGGCATATAC | To amplify IR7 without Linker between stem-loops |
| IR7∆Linker_R2 | | CTTCTTCTCCTTTTGATACCATAATATTACCCCCTTTTTATTTTTTATATATTGATCAACTACTTTGG |  |
| IR7∆Loop_F1 | | GAAATATACGTAAATCTCTGTTTATTGCCCGGCAGACATATATGCC | To amplify IR7 without Loop of stem-loop |
| IR7∆Loop_F2 | | TTTTAACATATCAATATCAATGAAATATACGTAAATCTCTG |  |
| IR7∆Loop_F3 | | GGAAGATCTCATACCTTTTAACATATCAATATCAATG |  |
| IR7∆stem_F | | CTTAAATTATAAAAATTAAATCTCATACTTAAATTATAAAAATTA | To amplify IR7 without stem regions of stem-loop |
| IR7∆Bubble_F1 | | CAATGAAATATACGTAAATCTCTGTTATTGCCCGGCAGACACAAATG | To amplify IR7 without Bubble of stem-loop |
| IR7∆Bubble_F2 | | GGAAGATCTCATACCTTTTAACATATCAATATCAATGAAATATACGTAAATC |  |
| IR10∆stem_F1 | | GTATTGATGCGTTGGACTATTAAAGATATATTTGTAATAGCTGAGTTCCCC | To amplify IR10 without stem regions of stem-loop |
| IR10∆stem_F2 | | GATCTTGGGGCGGCTGATGTCAACGGAGGTAAGAGTATTGATGCGTTGGACTAT |  |
| IR10∆stem_F3 | | GGAAGATCTTATCAATGACTTCCCGGTGGAAGATGATCTTGGGGCGGCTGATGTC |  |
| mcherry-R | | CGGAATTCTTATTTATAAAGTTCATCC | Downstream primer |
| For adding or deleting stem-loop of transcripts | | | |
| IR2_F | | GGAAGATCTTATTTAATACTATGACGCATATG | To add IR1-SL2 at downstream of IR2 |
| IR2::SL2_R1 | | TTATGACAGTAAAACTGCCATAAGTTCTTACCTTAATCCTTTTC |  |
| IR2::SL2_R2 | | CTTCTTCTCCTTTTGATACCATTTATGACAGTAAAACTGCCATAA |  |
| IR4::SL2_F | | GGAAGATCTCTTATGGCAGTTTTACTGTCATAAAATGAGTTTTTAATGCTTGCATTAC | To add IR1-SL2 at upstream of IR4 |
| fbfp-SL_F1 | | CAAGCCATAAAGTATGGAGATGATAAATGCAAAACTTC | To add stem-loop at upstream of the reporter gene *fbfp* |
| fbfp-SL_F2 | | CTAGCTAGCTTCCATACTTTATGGCATCAAGCCATAAAGTATGGAG |  |
| mcherry_R | | CGGAATTCTTATTTATAAAGTTCATCC | Downstream primer |
| For mutation of Type Ⅰ stem-loop | | | |
| IR1-SL1∆GC1_F1 | | ATCAAGCCATAAAGTATTAAAAAAACATATGAGCAGA | To delete GC1 of SL1 in IR1-SL1 |
| IR1-SL1∆GC1_F2 | | TTTAAATTATACTTTATGGCATCAAGCCATAAAGTATT |  |
| IR1-SL1∆GC1_F3 | | GGAAGATCTTTATTGAATTTAAATTATACTTTATGGC |  |
| IR1-SL1∆GC2_F1 | | ATCAAATAAAGTATGGAGTAAAAAAACATATGAGCAGA | To delete GC2 of SL1 in IR1-SL1 |
| IR1-SL1∆GC2_F2 | | TTCCATACTTTATATCAAATAAAGTATGGA |  |
| IR1-SL1∆GC2_F3 | | GGAAGACTTTATTGAATTTAAATTTTCCATACTTTATATCAAAT |  |
| IR1-SL1∆GC1∆GC2_F1 | | TTATACTTTATATCAAATAAAGTATTAAAAAAACATATGAGCAGATTTTAC | To delete GC1&GC2 of SL1 in IR1-SL1 |
| IR1-SL1∆GC1∆GC2_F1 | | GGAAGATCTTTATTGAATTTAAATTATACTTTATATCAAATAAAG |  |
| IR1-SL1-CCGG::GGCC_F1 | | ATCAAGCCATAAAGTATCCAGTAAAAAAACATATGAGCAGA | To mutate the order of GC1 pairs (CCGG) to GGCC in IR1-SL1 |
| IR1- SL1  ∆CCGG::GGCC_F2 | | TTGGATACTTTATGGCATCAAGCCATAAAGTATCCAG |  |
| IR1- SL1  ∆CCGG::GGCC_F3 | | GGAAGATCTTTATTGAATTTAAATTTTGGATACTTTATGGCATCAAG |  |
| IR1- SL1  ∆CCGG::CGCG_F1 | | ATCAAGCCATAAAGTATCGAGTAAAAAAACATATGAGCAGA | To mutate the order of GC1 pairs (CCGG) to CGCG in IR1-SL1 |
| IR1- SL1  ∆CCGG::CGCG_F2 | | TTCGATACTTTATGGCATCAAGCCATAAAGTATCGAG |  |
| IR1- SL1  ∆CCGG::CGCG_F3 | | GGAAGATCTTTATTGAATTTAAATTTTCGATACTTTATGGCATCAAG |  |
| IR1- SL1  ∆CCGG::GCGC_F1 | | ATCAAGCCATAAAGTATGCAGTAAAAAAACATATGAGCAGA | To mutate the order of GC1 pairs (CCGG) to GCGC in IR1-SL1 |
| IR1- SL1  ∆CCGG::GCGC_F2 | | TTGCATACTTTATGGCATCAAGCCATAAAGTATGCAG |  |
| IR1- SL1  ∆CCGG::GCGC_F3 | | GGAAGATCTTTATTGAATTTAAATTTTGCATACTTTATGGCATCAAG |  |
| IR5-SL1∆GC1_F1 | | ATTTGGAACAATAAATGCAACTAGTGCATTTATTGTTCCTATC | To delete GC1 of SL1 in IR5-SL1 |
| IR5-SL1∆GC1_F2 | | GGAAGATCTTTTTGAAGTTTTATTTGGAACAATAAATGCAACTAGTGCATTTATTGTTCCTATC |  |
| IR5-SL1∆GC2_F1 | | ATTTGGAACAATAGGAATAACTAGTATTCCTATTGTTCCTATC | To delete GC2 of SL1 in IR5-SL1 |
| IR5-SL1∆GC2_F2 | | GGAAGATCTTTTTGAAGTTTTATTTGGAACAATAGGAATAAC |  |
| IR5-SL1∆GC3_F1 | | ATTTGGAACAATAAATGCAACTAGTGCATTTATTGTTCCTATC | To delete GC3 of SL1 in IR5-SL1 |
| IR5-SL1∆GC3_F2 | | GGAAGATCTTTTTGAAGTTTTATTTGGAACAATA..AATGCAACTAGTGCATTTATTGTTCCTATC |  |
| IR5-SL1∆GC2∆GC3_F1 | | TTTTGAAGTTTTATTTGGAACAATAACTAGTATTGTTCCTATC | To delete GC1&GC2 of SL1 in IR5-SL1 |
| IR10-SL∆GC1::UA_F1 | | GTTGGACTATUUAGCAGTAAAGAGCTATTTGCTGCTAATAATAGCTGAGTTCCCCGG | To mutate GC1 of IR10-SL to UA |
| IR10-SL∆GC1::UA_F2 | | GCGGCTGATGTCAACGGAGGTAAGAGTATTGATGCGTTGGACTATUUAGCAGTAAAG |  |
| IR10-SL∆GC1::UA_F3 | | TATCAATGACTTCCCGGTGGAAGATGATCTTGGGGCGGCTGATGTCAACGGAGG |  |
| IR10-SL∆GC2::UA_F1 | | GCGTTGGACTATGCAGCAUUAAAGAGCTATTTAATGCTGCTAATAGCTGAGTTCCCCG | To mutate GC2 of IR10-SL to UA |
| IR10-SL∆GC2::UA_F2 | | GGGGCGGCTGATGTCAACGGAGGTAAGAGTATTGATGCGTTGGACTATGCAGC |  |
| IR10-SL∆GC2::UA_F3 | | TATCAATGACTTCCCGGTGGAAGATGATCTTGGGGCGGCTGATGTCAACGG |  |
| mcherry_R | | CGGAATTCTTATTTATAAAGTTCATCC | Downstream primer |
| For mutation of Type Ⅱ stem-loop | | | |
| IR2-SLGC1::UA_F1 | | GATTAAATTACTCATTCTTGTACTGTAAGGGCTTATGAGTTACAAAG | To mutate GC1 of IR2-SL to UA |
| IR2-SL∆GC1::UA_F2 | | GTAACCTTAAAGTCCTTACAGTATTTGGTTTGATTAAATTACTCATTC |  |
| IR2-SL∆GC1::UA_F3 | | GGAAGATCTTATTTAATACTATGACGCATATGTAACCTTAAAGTCCUUACAGTATTTGG |  |
| IR2-SL∆GC2::UA_F1 | | GATTAAATTACTCATTCTTGTACTGTAAGGAATTATGAGTTACAAAG | To mutate GC2 of IR2-SL to UA |
| IR2-SL∆GC2::UA_F2 | | GTAACCTTAAAUUCCUUACAGTATTTGGTTTGATTAAATTACTCATTC |  |
| IR2-SL∆GC2::UA_F3 | | GGAAGATCTTATTTAATACTATGACGCATATGTAACCTTAAAUUCCUUACAGTATTTGG |  |
| IR2-SL∆GC3::UA_F1 | | GATTAAATTACTCATTCTTGTACTGTAAAAAATTATGAGTTACAAAG | To mutate GC3 of IR2-SL to UA |
| IR2-SL∆GC3::UA_F2 | | GTAACCTTAAAUUUUUUACAGTATTTGGTTTGATTAAATTACTCATTC |  |
| IR2-SL∆GC3::UA_F3 | | GGAAGATCTTATTTAATACTATGACGCATATGTAACCTTAAAUUUUUUACAGTATTTGG |  |
| mcherry_R | | CGGAATTCTTATTTATAAAGTTCATCC | Downstream primer |
| For mutation stem-loop of IR4 | | | |
| IR4∆SL1 | | GGAAGATCTTTTTTAATGCTTGCATTACTAAATG | To delete SL1 in IR4 |
| IR4∆SL2-28-34_F | | TAGAAGTTCAGTTTGGAAGTTTAATGAGGCTTGCATTACTAAATG | To delete 5’ single- strand of AT-pair region in IR4 |
| IR4∆SL2-59-65_F | | GCTTGCATTACTAAATGTAAGCATAAAAAATTTTAC | To delete 3’ single- strand of AT-pair region in IR4 |
| IR4∆SL1::SLx_F | | GGAAGATCTCTTATGGCAGTTTTACTGTCATAAAATGAGTTTTTAATGCTTGCATTAC | To replace SL1-IR4 with SLx |
| IR4∆SL2-36-57::IR1_SL1_F1 | | GGCATCAAGCCATAAAGTATGGAGTTAAAAAATAAAAAATTTTACTAGGAGG | To replace upper part of SL2-IR4 with SL1-IR1 |
| IR4∆SL2-36-57::IR1_SL1_F2 | | GGAAGTTTAATGAGTTTTTAATTCCATACTTTATGGCATCAAGCCATAAAGTATGG |  |
| IR4∆SL2-36-57::IR1_SL1_F3 | | GGAAGATCTTAGAAGTTCAGTTTGGAAGTTTAATGAGTTTTTAATTCC |  |
| IR4∆SL2::IR1_SL1_F1 | | CAAGCCATAAAGTATGGAGTTTAAAAATAAAAAAATTTTACTAGGAGGTAAAT | To replace SL2-IR4 with SL1-IR1 |
| IR4∆SL2::IR1_SL1_F2 | | GAGTTTTTAATTTCCATACTTTATGGCATCAAGCCATAAAGTATGGAGTTT |  |
| IR4∆SL2::IR1_SL1_F3 | | GGAAGATCTTAGAAGTTCAGTTTGGAAGTTTAATGAGTTTTTAATTTCCATAC |  |
| mcherry_R | | CGGAATTCTTATTTATAAAGTTCATCC | Downstream primer |
| For cloning of modified stem-loops | | | |
| SLx_F | | GGAAGATCTTTATGGCAGTTTTACTGTCATAAATGGTATCAAAAGGAGAAGAAG | To amplify SLx |
| SLx+GC1_F | | GGAAGATCTCCTTATGGCAGTTTTACTGTCATAAGGATGGTATCAAAAGGAGAAGAAG | To amplify Slx with GC1 added on the bottom of stem |
| SLx+GC1+GC2_F1 | | GCTTTTAGCCCTGTCATAAAGGATGGTATCAAAAGGAGAAGAAG | To amplify Slx with GC1/GC2 added on the bottom and top of stem |
| SLx+GC1+GC2_F2 | | GGAAGATCTCCTTATGGCAGGGCTTTTAGCCCTGTCATAAGG |  |
| SLx+UA1_F | | GGAAGATCTUUTTATGGCAGTTTTACTGTCATAAAAATGGTATCAAAAGGAGAAGAAG | To amplify Slx with UA1 added on the bottom of stem |
| SLx+UA1+UA2_F1 | | UUTTATGGCAGUUTTTTAAACTGTCATAAAAATGGTATCAAAAGGAGAAGAAG | To amplify Slx with UA1/UA2 added on the bottom and top of stem |
| SLx+UA1+UA2_F2 | | GGAAGATCTUUTTATGGCAGUUTTTTAAACTGTCATAAAAATGGTATCAAAAGGAGAAGAAG |  |
| SLy_F | | GGAAGATCTCCCGGCATCAAGCCGGGAAAATTTAGGAGGTTAGTTAGAATG | To amplify SLy |
| Sly+AT1_F | | GGAAGATCTCCCGGCAATCAATGCCGGGAAAATTTAGGAGGTTAGTTAGAATG | To amplify Sly with AT1 added on the top of stem |
| Sly+AT1+TA2_F | | GGAAGATCTTTCCCGGCAATCAATGCCGGGAAAAAATTTAGGAGGTTAGTTAGAATGTG | To amplify Sly with AT1/TA2 added on the top and bottom of stem |
| mcherry_R | | CGGAATTCTTATTTATAAAGTTCATCC | Downstream primer |
| For cloning of reporter system | | | |
| mcherry_F | | GAAGATCTATGGTATCAAAAGGAGAAGAAG | To amplify the DNA fragment of *mcherry* |
| mcherry_R | | CGGAATTCTTATTTATAAAGTTCATCCATTCCTCC |  |
| fbfp_F | | CTAGCTAGCATGATAAATGCAAAACTTCTTCAGC | To amplify the DNA fragment of *fbfp* |
| fbfp_R | | CGGGATCCTTAATGTTTTGCCTGACCCTG |  |
| For Northern botting | | | |
| fbfp_probe | | GCTGAAGAAGTTTTGCATTTATCAT | To probe gene *fbfp* |
| mcherry_probe | | CTCGTCTTCCTCTGTAAGTTGTCCTCCT | To probe gene *mcherry* |
| cipC_F | | ATGCGTAAAAAGTCTTTAGCATTTTTG | To amplify *cipC* gene probes |
| cipC _R | | GTTCTGCTACTTCTGCTTCAAGGGAAGACTT |  |
| fbfp_R | | CTGTCTTCTAAGTTCTGCTACTTCTGCTTCAAG |  |
| For primer extension analyses | | | |
| Mcherry_P1 | | CTTCTTCTATTATACCGTTA | To target 5’ end of processed transcripts |
| For Real-time quantitative RT-PCR(qPCR) | | | |
| mcherry_F | | ATTTTCCTTCAGATGGACCTGT | Intragenic region of *mcherry*, qRT-PCR |
| mcherry_R | | ATTATATGCTCCAGGAAGCTGT |  |
| fbfp_F | | CATGATCAGCCTGGTATAGC | Intragenic region of *fbfp*, qRT-PCR |
| fbfp_R | | TCTTCTGCAAATACCTGTGCT |  |
| Ccel_RS01560_F | | AGATACTAAGCTCGGTCCTGA | Intragenic region of *Ccel_RS01560*, qRT-PCR |
| Ccel_RS01560_R | | CACCAAAGATCGCTCTAAGCA |  |
| For generating and validating the mutant | | | |
| Ccel_RS08950_IBS | | CCGCTCGAGATAATTATCCTTAGTTGCCTTAGC AGTGCGCCCAGATAGGGTG | To construct a targeting region for *Ccel_RS08950* |
| Ccel_RS08950_EBS | | TGAACGCAAGTTTCTAATTTCGATTGCAACTCG ATAGAGGAAAGTGTCT |  |
| Ccel_RS08950_EBS1d | | CAGATTGTACAAATGTGGTGATAACAGATAAG TCTTAGCAATTAACTTACCTTTCTTTGT |  |
| EBS universal primer | | CGAAATTAGAAACTTGCGTTCAGTAAAC |  |
| Ccel_RS08950_F | | GTGTCAAAAAGCAAAAAAA | To validate the mutant of *Ccel_RS08950* |
| Ccel_RS08950_R | | TTATATTTCCATTATAATAGGT |  |

Table S3. Sequences of IRs in the *cip-cel* operon

| IRs | | Sequence (5’-3’) | Description |
| --- | --- | --- | --- |
| IR1 | TTATTGAATTTAAATTTTCCATACTTTATGGCATCAAGCCATAAAGTATGGAGTAAAAAAACATATGAGCAGATTTTACTCTGCCATAAAAATTCAAAGAATAAGGAAGGTGTAAA | | The intergenic region sequence of *cipC*-*cel48F* (IR1) |
| IR2 | TATTTAATACTATGACGCATATGTAACCTTAAAGTCCGGACAGTATTTGGTTTGATTAAATTACTCATTCTTGTACTGTCCGGGCTTATGAGTTACAAAGAAAAAAAGAAAAGGATTAAGGTAAGAAC | | The intergenic region sequence of *cel48F*-*cel8C* (IR2) |
| IR3 | AATAAATAAAAATAATTGAGTGAGCATCTCAGGTTAAATTTGTCTTAAAAATGTTTAAATTTAATTTTAGGGAGTGATGGCAAG | | The intergenic region sequence of *cel8C*-*cel9G* (IR3) |
| IR4 | TAGAAGTTCAGTTTGGAAGTTTAATGAGTTTTTAATGCTTGCATTACTAAATGTAAGCTTTAAAAAATAAAAAATTTTACTAGGAGGTAAAT | | The intergenic region sequence of *cel9G*-*cel9E* (IR4) |
| IR5 | TTTTGAAGTTTTATTTGGAACAATAGGAATGCAACTAGTGCATTCCTATTGTTCCTATCCTATTAGTTTCAGATTTATGATTTGAACGAATTTGTTATTTCCTTTAATTAAAATGTTTATAGTAATTTTTTAGTAATAAAATATATTGAATAAATAAGGAGAAAAGCC | | The intergenic region sequence of *cel9E*-*orfX* (IR5) |
| IR6 | GAACTCAAGAAGATTAATCCTGTATTCACAGAGGGTGAGATAACAGTTAAATAACTGATTCATGCATGGAGTGTGTAAAT | | The intergenic region sequence of *orfX*-*cel9H* (IR6) |
| IR7 | CATACCTTTTAACATATCAATATCAATGAAATATACGTAAATCTCTGTTTATTGCCCGGCAGACACAAATGGCATAAAGATTGTATTTTGCCAGTGTATATGCCGGGTAATGACAATACTTAAATTATAAAAATTATTATTCCAAAGTAGTTGATCAATATATAAAAAATAAAAAGGGGGTAATATT | | The intergenic region sequence of *cel9H*-*cel9J* (IR7) |
| IR8 | TGCTCTTGACCTTGCTTTATTAAAGAAGACTCTACTTGGTTAATACAGCCTGTTAATTCGAAAAGTAAGGAGGATTTGCT | | The intergenic region sequence of *cel9J* -*Man5K* (IR8) |
| IR9 | ATAATTTATTACAGTGTATTTGACCTGTCCGTTAAAAACGGTTCTTTAAGTCGGACAGAAATTTTTAAATAAAAAAGGGAGGGATAGTT | | The intergenic region sequence of *Man5K*-*cel9M* (IR9) |
| IR10 | TATCAATGACTTCCCGGTGGAAGATGATCTTGGGGCGGCTGATGTCAACGGAGGTAAGAGTATTGATGCGTTGGACTATGCAGCAGTAAAGAGCTATTTGCTGCTGCTAATAGCTGAGTTCCCCGGAAGGTAATTTCAGGCAGTTTTAGTAAAAGGAGGGAAAAGT | | The intergenic region sequence of *cel9M* -*rgl11Y* (IR10) |
| IR11 | CACTAGACTTGGCATTACTAAAAGCAAGTTTGCTTTCGTAGTGTTTAGGAAAGGTTATTAAACAAAAAGGAGAAGAACCT | | The intergenic region sequence of *rgl11Y* -*cel5N* (IR11) |

Reference

Cui, G. Z., et al. Targeted gene engineering in Clostridium cellulolyticum H10 without methylation. *J Microbiol Methods* **2012**, 89, 201-208.

Shao, L., et al. Targeted gene disruption by use of a group II intron (targetron) vector in Clostridium acetobutylicum. *Cell Res* **2007**, 17, 963-965.
